# Multi-depth switching by triple wavefront modulation of quarter-waveplate geometric phase lenses for vergence-accommodation-matching extended reality

**DOI:** 10.1038/s41377-025-02026-2

**Published:** 2025-09-19

**Authors:** Jung-Yeop Shin, Jae-Won Lee, Hafiz Saad Khaliq, Erkhembaatar Dashdavaa, Munkh-Uchral Erdenebat, Min-Seok Kim, Jin-Hyeok Seo, Young-Min Cho, Hak-Rin Kim

**Affiliations:** 1 School of Electronic and Electrical Engineering, Kyungpook National University, Daegu, Republic of Korea; 2 Center for Semiconductor-Specialized University, Kyungpook National University, Daegu, Republic of Korea; 3 School of Electronics Engineering, Kyungpook National University, Daegu, Republic of Korea

**Keywords:** Optoelectronic devices and components, Displays, Liquid crystals

## Abstract

We present a novel approach to resolving the vergence–accommodation conflict (VAC) in extended reality (XR) optics by introducing a quarter-waveplate (QWP) geometric phase lens (GPL) capable of triple wavefront modulation—focusing, defocusing, and non-modulating at infinity. This polarization-driven behavior is interpreted using contour trajectories on the Poincaré sphere and compared against conventional half-waveplate (HWP) GPLs. Leveraging this property, we propose a bi-stacked QWP GPL module that enables nine distinct varifocal states through polarization-controlled input selection and output filtering. In contrast, HWP-based modules under equivalent stacking conditions are limited to four focal states. The QWP GPL module supports a compact varifocal system spanning a continuous depth range from 24.27 cm to infinity, with a 0.3-diopter interval aligned with the human visual comfort zone. Importantly, the number of representable focal depths scales as 3^n^ for *n* stacked layers, offering a (1.5)^n^-fold improvement over the 2^n^ scaling of HWP systems. This enables finer depth transitions using fewer lens units while retaining both compactness and optical modularity, establishing a depth-switchable imaging platform that enhances visual comfort and depth fidelity in next-generation XR display systems.

## Abstract (web summary)

Schematic representation of nine-depth accommodative virtual images spanning the full perceivable depth range without inducing vergence–accommodation conflict (VAC), achieved using a bi-stacked quarter-waveplate geometric phase lens (QWP GPL) module. A conceptual illustration of VAC-resolved XR operation is also provided, demonstrating the ideal alignment between virtual image planes and real-world scene depths under intended system behavior.

## Graphical abstract

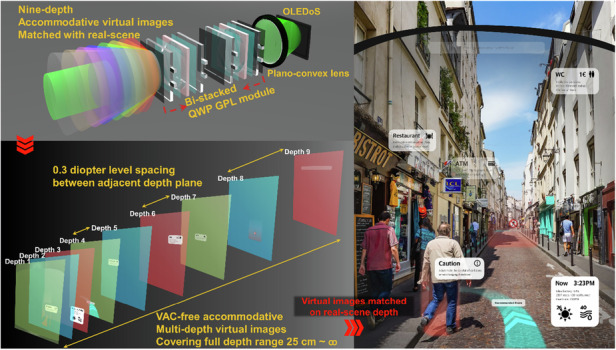

## Introduction

Extended reality (XR) systems are rapidly evolving into wearable platforms that deliver immersive, context-aware visual experiences by seamlessly integrating virtual content with the physical environment^1–7^. A core requirement of XR optics is to render digital imagery with spatial precision, particularly in interactive scenarios occurring at hand-reachable distances, typically encompassing depth ranges below 30–50 cm. These near-field conditions are especially critical, as the human visual system is most sensitive to inconsistencies in vergence and accommodation cues within this short range. When virtual focal planes fail to align with the depth of real-world objects, users experience pronounced vergence–accommodation conflict (VAC)—a physiological mismatch between the convergence of the eyes and their focal accommodation—which leads to serious visual fatigue, discomfort, and ultimately, reduced usability of XR devices^8–12^. This challenge is further intensified by the requirement to support a wide depth range—from near-field interactions to optical infinity—within the constraints of compact, wearable XR systems^10,13,14^. To ensure visual comfort across this range, depth-switchable optics must generate multiple, or ideally continuous, virtual focal planes spaced within the eye’s accommodation comfort zone. In near-field viewing conditions, this spacing criterion becomes especially stringent, often requiring dioptric intervals as fine as 0.3 diopters or less to remain perceptually seamless^15^. However, achieving such high-resolution varifocal transitions without compromising the system’s form factor or introducing mechanical complexity remains a fundamental design constraint in XR optics.

To overcome these limitations, various approaches such as deep learning-based holographic displays^16,17^ and light field displays^18^ have been investigated. Among various approaches that aims to resolve VAC issue in XR device, a range of polarization-dependent optical strategies especially has been actively explored^1,4,10,13,19–28^. These systems modulate wavefronts via polarization-state control, exploiting the human eye’s insensitivity to polarization. A major advantage is that each element can be implemented as a flat, ultra-thin optical layer, enabling stacking into modular structures without compromising the overall device form factor. Varifocal operation is achieved by switching the polarization state between layers, allowing dynamic focal control without the need for mechanical movement. Technologies in this category include LC lenses^1,4,10,29–33^, meta-optic lenses^34–40^, and LC-based geometric phase lenses (GPLs)^20–22,41,42^. For instance, meta-lenses offer subwavelength-resolution phase control via nanostructured designs and are considered promising candidates for ultrathin optics. However, their current limitations—including fabrication cost, aperture scalability, and chromatic aberrations—continue to hinder their practical adoption in XR systems. In contrast, LC-based GP devices provide high efficiency, broadband performance across the visible spectrum, large-area scalability, and low fabrication cost. Their operating principle relies on the spatial distribution of the optic axis orientation in anisotropic media, where the local phase retardation is typically defined by the half-waveplate (HWP) condition. This approach enables precise phase control across flat substrates without requiring bulk curvature or thickness, similar to the operating principles of meta-optics. As a result, GP holographic (GPH) optics has enabled a wide range of flat optic components beyond conventional lenses—including vortex plates, prisms, beam deflectors, and diffraction gratings—highlighting its utility across diverse wavefront-shaping applications^21^. The flexibility and scalability of LC-based GPH thus positions it as a highly promising platform not only for varifocal XR optics, but also for next-generation flat optical systems.

Within various implementations of GPH optics, HWP-based GPLs have been widely adopted for varifocal imaging due to their binary wavefront switching between positive and negative focal powers. By stacking *n* layers of HWP GPLs with polarization-switching elements, up to 2^n^ discrete focal states can be realized^10,13,19^. However, as the number of required focal depths or total depth range increases, additional layers must be incorporated, leading to increased module thickness and structural complexity. Moreover, with each additional layer, cumulative optical aberrations—such as wavefront distortion and focal shift—become more significant due to interlayer misalignments and birefringent dispersion. These limitations pose a fundamental barrier to the integration of HWP-based GPL architectures in compact, high-performance XR display systems. To address these trade-offs, an alternative approach is needed—one that enables more than two wavefront modulations per unit GPL without incurring additional structural burden. A system capable of supporting multi-state wavefront modulation in a single-layered configuration could substantially improve varifocal resolution, reduce device thickness, and mitigate wavefront degradation. This necessity motivates the development of a new class of LC-based GPL architecture optimized for scalable, high-density depth switching in next-generation, thin-form XR optics.

## Results

### Geometric phase modulations under half- and quarter-waveplate conditions

**Fig. 1:**
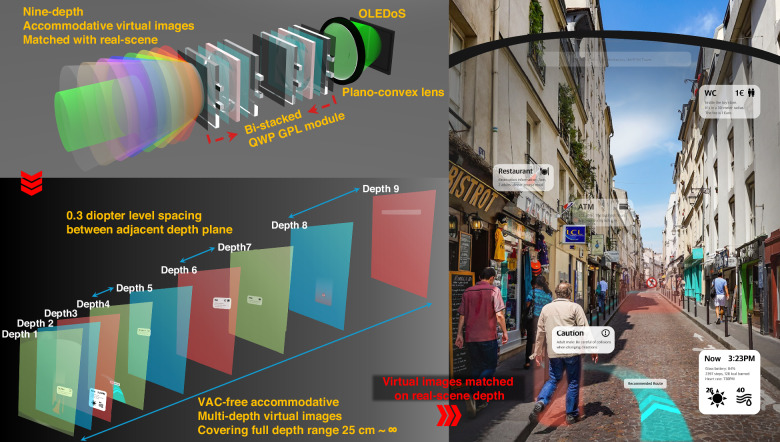
Schematic representation of nine-depth accommodative XR device using bi-stacked QWP GPL module. Schematic representation of nine-depth accommodative virtual images spanning the full perceivable depth range without inducing vergence–accommodation conflict (VAC), achieved using a bi-stacked quarter-waveplate geometric phase lens (QWP GPL) module. A conceptual illustration of VAC-resolved XR operation is also provided, demonstrating the ideal alignment between virtual image planes and real-world scene depths under intended system behavior

To address the intrinsic depth-scalability limitations of conventional HWP-based GPL architectures, we introduce a varifocal design based on a quarter-waveplate (QWP) GPH approach. While HWP GPLs support only binary wavefront switching, the proposed QWP-based structure enables triple-state modulation—focusing, defocusing, and non-focusing—within a single optical layer. This principle allows up to 3^n^ discrete focal states through *n*-layer stacking, significantly enhancing depth resolution while minimizing system thickness and complexity. We experimentally implemented a bi-stacked QWP GPL module and integrated it into an XR imaging system, achieving nine switchable virtual image planes spaced at 0.3 diopter intervals and spanning from 24.27 cm to optical infinity. As shown conceptually in Fig. 1, the system achieves VAC-resolved depth placement across the perceptual range while maintaining a compact and wearable optical configuration.

**Fig. 2:**
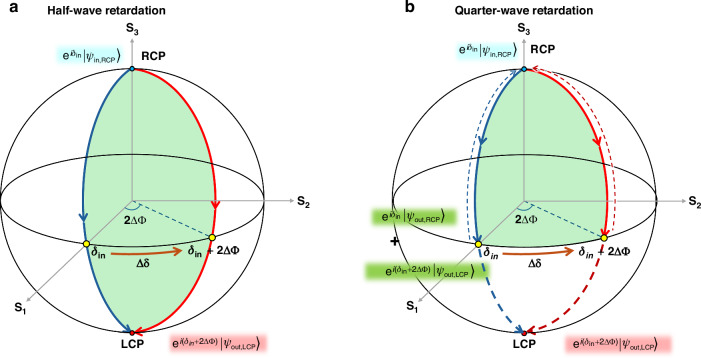
Contour trajectories on the Poincaré sphere of an anisotropic medium under HWP versus QWP condition. Contour trajectories on the Poincaré sphere formed by two optical axes of an anisotropic medium under **a** half-waveplate and **b** quarter-waveplate conditions

When an anisotropic medium with a spatially varying optic axis distribution *Φ*(*x*,*y*) is formed on a planar surface, light traversing the birefringent layer accumulates a spatially dependent phase shift *δ*(*x*, *y*). The relative phase difference *δ*, induced by a local optic axis rotation Δ*Φ*, corresponds to the geometric phase (GP), or Pancharatnam–Berry phase^43,44^. This GP modulation effect can be visualized on the Poincaré sphere, where the contour trajectory subtends a solid angle Ω, yielding a phase shift of Δδ = Ω/2. Figure 2 shows how the GP modulation arises under HWP and QWP conditions^45–47^. For right-handed circularly polarized (RCP) incident light described by ${e}^{i{\delta }_{{in}}}|{\psi }_{{in},{RCP}}\rangle$, the reference and rotated optic axes define a trajectory enclosing a solid angle on the sphere. Under the HWP condition (Γ=π, Fig. 2a), the RCP state undergoes half-wave retardation and is converted into left-handed circular polarization (LCP), accumulating a phase shift of Δ*δ* = 2Δ*Φ*. The output wavefront is thus described as ${e}^{i\left({\delta }_{{in}}+2\Delta \Phi \right)}|{\psi }_{{out},{LCP}}\rangle$, indicating spatially varying phase delay patterns. The modulation efficiency *η* for the non-modulated (0^th^ order) and modulated ( ±1^st^ order) terms is defined as:

1 $${\eta }_{0}={\cos }^{2}\left(\frac{\Gamma }{2}\right)$$

2 $${\eta }_{\pm 1}=\frac{1}{2}\left[1\mp {S}_{3}\right]{\sin }^{2}\left(\frac{\Gamma }{2}\right)$$

where *S*_3_ is the Stokes parameter for incident circular polarization. GPH layers have been typically optimized at Γ=π to maximize ±1^st^-order efficiency and suppress the 0^th^-order term.

In contrast, the QWP condition (Γ=π/2), proposed in this work, yields different modulation behavior. As shown in Fig. 2b, the RCP input light is converted into linear polarization, which can be decomposed into two orthogonal circular components of equal amplitude for further analysis^48,49^. Jones matrix analysis gives:

3 $$\left(\begin{array}{cc}\cos \left(\Phi \right) & -\sin \left(\Phi \right)\\ \sin \left(\Phi \right) & \cos \left(\Phi \right)\end{array}\right)\left(\begin{array}{cc}{e}^{-i\frac{{\boldsymbol{\pi }}}{4}} & 0\\ 0 & {e}^{+i\frac{{\boldsymbol{\pi }}}{4}}\end{array}\right)\left(\begin{array}{cc}\cos \left(\Phi \right) & \sin \left(\Phi \right)\\ -\sin \left(\Phi \right) & \cos \left(\Phi \right)\end{array}\right)\left(\begin{array}{c}1\\ \pm i\end{array}\right)=\frac{\sqrt{2}}{2}\left\{\left(\begin{array}{c}1\\ \pm i\end{array}\right)+\left(\begin{array}{c}1\\ \mp i\end{array}\right){e}^{i\left(-\frac{{\boldsymbol{\pi }}}{2}\pm 2\Phi \right)}\right\}$$

indicating a superposition of a non-modulated 0^th^-order term and a modulated ±1^st^-order term. On the Poincaré sphere, the 0^th^-order component traces a closed contour with zero enclosed area—yielding no geometric phase shift—while the modulated component encloses a finite solid angle, producing Δ*δ* = 2Δ*Φ* as in the HWP-based GPH case. Crucially, under the QWP conditions, both wavefronts emerge simultaneously with equal intensity. This intrinsic decomposition yields a 1:1 intensity ratio between modulated and non-modulated terms, forming the basis of the triple-state modulation unique to QWP-based GPH optics. In the context of the proposed GPL, this enables selective generation of focusing, defocusing, or neutral (non-modulated) wavefronts through appropriate polarization control and selection.

### Quarter-waveplate geometric phase lens

**Fig. 3:**
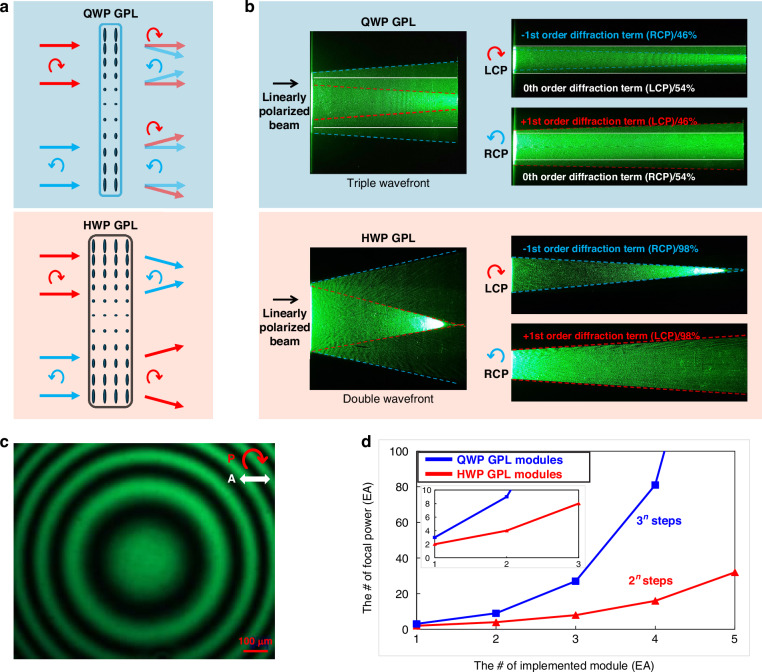
QWP versus HWP GPLs operation. **a** Schematic illustration of wavefront modulation effects and **b** experimental beam propagation results for both HWP and QWP GPLs under different incident polarization states. **c** Polarization optical microscopy image of a fabricated QWP GPL, obtained using a circular polarizer at the input and a linear analyzer at the output. **d** Number of representable varifocal steps as a function of the number of stacked HWP and QWP GPL module configurations

The application of GP modulation under QWP and HWP conditions enables the realization of polarization-controlled varifocal functionality in GPLs. The optic axis distribution of the GPL is defined by a spatial lens phase function:

4 $$\Phi \left({\rm{x}},{\rm{y}}\right)=\frac{2\pi }{{\lambda }_{d}}\left(f-\sqrt{{r}^{2}-{f}^{2}}\right)$$

where ${\lambda }_{d}$ is the design wavelength, $f$ is the focal length, and $r$ denotes the radial coordinates. The varifocal modulation behavior differs fundamentally between QWP- and HWP-based GPLs. As shown in Fig. 3a, HWP-based GPLs support binary focal states of ±*f* by switching between ±1^st^-order modulations, functioning as concave or convex lenses depending on the handedness of the incident circular polarization. In contrast, QWP-based GPLs offer a third modulation mode by simultaneously producing both modulated ( ±1^st^ order) and unmodulated (0^th^ order) wavefronts. This triple-state response allows switching among focusing, defocusing, and neutral (non-modulated) transmission based solely on polarization state control.

To experimentally validate these behaviors, 1-inch-diameter QWP and HWP GPLs were fabricated using photoalignment-based liquid crystal processing and tested under RCP, LCP, and linear polarization conditions. Figure 3b presents the beam propagation patterns obtained with a 532 nm collimated laser. The HWP GPL exhibited a high ±1^st^-order modulation efficiency of 94%, with polarization-selective switching between focusing and defocusing states. By comparison, the QWP GPL showed 48% efficiency per ±1^st^ order, accompanied by a simultaneous 0^th^-order component. Notably, under linear polarization, the QWP GPL generated all three wavefronts, verifying its triple-state capability. Further confirmation of the optic axis alignment and phase distribution in the QWP GPL was obtained via polarization optical microscopy. Figure 3c shows the observed LC alignment pattern using a circularly polarized incident beam and a linear analyzer, confirming the expected azimuthal and radial variation in optic axis orientation.

Beyond individual lens behavior, stacking QWP GPLs in modular configurations significantly expands varifocal functionality. With integrated polarization control and selection layers, an *n*-layer QWP GPL module can support up to 3^n^ discrete focal states. This scalability, illustrated in Fig. 3d, contrasts with HWP GPL modules, which are limited to 2^n^ transitions. For example, a bi-stacked QWP GPL module enables nine focal states, while an equivalent HWP module yields only four. This 1.5^n^-fold increase in focal transitions per module count highlights the superior scalability of QWP GPLs, enabling finer depth resolution with fewer optical layers and reduced structural complexity.

### Multi-step varifocal switching in Bi-stacked QWP GPL module

**Fig. 4:**
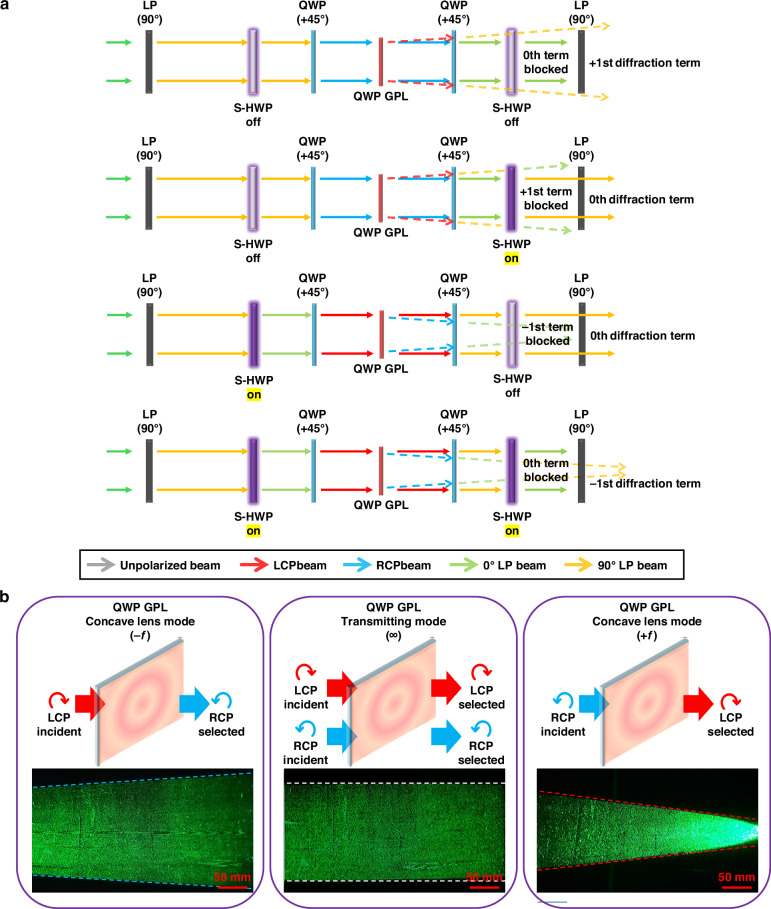
Polarization control and selective wavefront modulation in a QWP GPL module. **a** Schematic of a single QWP GPL module showing the polarization control and selection layer configuration, along with the associated wavefront modulation functions under switching operation. **b** Captured beam propagation images corresponding to different wavefront modulation states

To realize multi-step varifocal operation based on the triple-wavefront modulation of the QWP GPL, a compact optical module was configured with the following layer sequence: switchable half-waveplate (S-HWP), QWP film, QWP GPL, QWP film, S-HWP, and a linear polarizer (LP). This configuration electrically modulates wavefronts via polarization-state switching, eliminating the need for mechanical movement. The S-HWP, implemented using an electrically controlled birefringence (ECB)-mode LC cell (X-FPM(L)-AR, LC-Tec Displays AB) and driven by an external controller (LCC-230, LC-Tec Displays AB), enables two-step retardation control (0 and π). The measured switching speeds—0.23 ms (field-on) and 1.04 ms (field-off)—are much faster than the typical latency of eye-tracking systems ( ~3 ms), supporting seamless integration for real-time XR display updates (see Supplementary Section 1). An LP at the module input ensures 90° linear polarization, which the first S-HWP modulates between 0° and 90°, depending on the driving voltage. The following QWP films, aligned at 45°, convert the linear input into either RCP or LCP states, enabling polarization-dependent focus-tunable operation of the QWP GPL. A second polarization control unit—comprising a QWP, S-HWP, and LP—at the module output acts as a polarization selector, allowing the selective extraction of one of the two orthogonal wavefront components co-generated by the QWP GPL. Figure 4a illustrates the evolution of polarization and its corresponding ray modulations through each layer of the module. This architecture supports the generation of three discrete focal powers—+*f*, ∞, and −*f*—under voltage control alone. The beam propagation results shown in Fig. 4b experimentally confirm the selective modulation of these wavefront states. For comparison, the depth-switching behavior of a conventional HWP GPL module based on 2^n^ focal transitions is described in Supplementary Section 2.

### Optic design for VAC-free varifocal XR imaging system

**Fig. 5:**
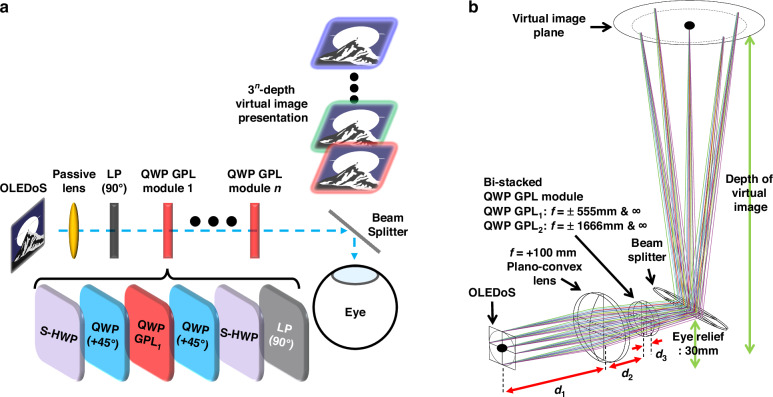
Optical design of a bi-stacked QWP GPL–based varifocal XR system. **a** Schematic of a 3^*n*^-depth varifocal beamsplitter-combiner-based XR imaging system implemented using stacked QWP GPL modules. **b** Zemax OpticStudio ray-tracing simulation model used to design optimal switchable depth planes for resolving vergence–accommodation conflict (VAC) in XR displays, realized with bi-stacked QWP GPL–based focus-switchable lens assemblies

As shown in Fig. 5a, the proposed bi-stacked QWP GPL module was integrated into an XR imaging system to enable polarization-controlled selection among 3^n^ virtual image depths. The optical setup consists of an OLEDoS display panel, a plano-convex lens (*f* = +10 cm), the bi-stacked QWP GPL module, and a beam combiner. Figure 5b defines the key spacing parameters: *d*₁ (display-to-lens), *d*₂ (lens-to-QWP GPL₁), and *d*₃ (between QWP GPLs). The virtual image distance formed by the plano-convex lens alone is given by:

5 $$\frac{1}{{v}_{{passive}}}=\frac{1}{{f}_{{passive}}}-\frac{1}{{d}_{1}}$$

Successive passage through QWP GPL_1_ and QWP GPL_2_ modifies this virtual image plane as follows:

6 $$\frac{1}{{v}_{{{QWP\; GPL}}_{1}}}=\frac{1}{{v}_{{passive}}+{d}_{2}}-\frac{1}{{f}_{{{QWP\; GPL}}_{1}}}$$

7 $$\frac{1}{{v}_{{{QWP\; GPL}}_{2}}}=\frac{1}{{v}_{{{QWP\; GPL}}_{1}}+{d}_{3}}-\frac{1}{{f}_{{{QWP\; GPL}}_{2}}}$$

For practical cases where *d*_2_ and *d*_3_ are small compared to *v*_*passive*_ and *v*_*QWP GPL1*_, the combined virtual image distance is approximated as:

8 $$\frac{1}{v(k)}\approx \frac{1}{{v}_{{passive}}}-\frac{1}{{f}_{{{QWP\; GPL}}_{1}}}-\frac{1}{{f}_{{{QWP\; GPL}}_{2}}}$$

To satisfy the VAC-free condition within a 0.3 D comfort zone, the ideal diopter distribution for *N* virtual image depths can be expressed as:

9 $$\displaystyle\frac{1}{{v}_{{ideal}}\left(k\right)}=[\left(2N+1\left)\right.-2k\right)]\cdot \left({\rm{comfort}}\; {\rm{zone}}\; {\rm{limit}}\right),{\rm{where}}\left(1\le k\le N\right)$$

Each QWP GPL supports focal states of +*f*, ∞, and −*f*. To achieve nine discrete focal depths (*N* = 9), focal lengths are assigned per lens as follows:

10 $$\begin{array}{l}{{f}_{{{QWP\; GPL}}_{1}}\left(k\right)=\left\{\begin{array}{l}+{f}_{{{QWP\; GPL}}_{1}}\left(k\le 3\right)\\\qquad\infty\qquad\;\, \left(3 < k\le 6\right)\\ -{f}_{{{QWP\; GPL}}_{1}}\left(k > 6\right)\end{array}\right.}\\{{f}_{{{QWP\; GPL}}_{2}}\left(k\right)=\left\{\begin{array}{l}+{f}_{{{QWP\; GPL}}_{2}}\left(k\,\mathrm{mod}\,3=1\right)\\\qquad\;\,\infty\qquad\left(k\,\mathrm{mod}\,3=2\right)\\ -{f}_{{{QWP\; GPL}}_{2}}\left(k\,\mathrm{mod}\,3=0\right)\end{array}\right.}\end{array}$$

At depth 5 (*k* = 5), both QWP GPLs are in ∞ states, leaving the focal distance determined solely by the passive lens, yielding:

11 $$\frac{1}{v\left(k=5\right)}=\frac{1}{{v}_{{passive}}}=\frac{1}{{f}_{{passive}}}-\frac{1}{{d}_{1}}=(2N-9)\cdot \left({\rm{comfort}}\;{\rm{zone}}\;{\rm{limit}}\right)$$

To maintain uniform 0.3 D spacing, the dioptric difference between adjacent depths must be:

12 $$\frac{1}{v\left(k\right)}-\frac{1}{v\left(k+1\right)}=2\cdot \left({\rm{comfort}}\; {\rm{zone}}\; {\rm{limit}}\right)\approx \frac{1}{{f}_{{{QWP\; GPL}}_{2}}}\approx \frac{1}{{f}_{{{QWP\; GPL}}_{1}}}-\frac{2}{{f}_{{{QWP\; GPL}}_{2}}}$$

**Fig. 6:**
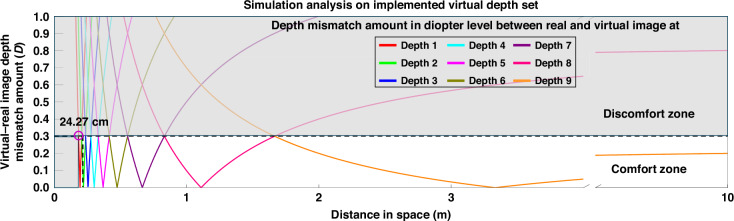
Depth mismatch curves across nine depth planes, expressed in diopters. Depth mismatch curves in diopters between the virtual image plane and the real-world scene plane, derived from simulation analysis of the nine-depth varifocal optical system implemented using a bi-stacked QWP GPL module

The optimized parameters were: *d*_1_ = 76.59 mm, *d*_2_ = 25.76 mm, *d*_3_ = 3.5 mm, *f*_*QWP GPL1*_ = ±555.56 mm ( ±1.8 D and 0 D), and *f*_*QWP GPL2*_ = ±1666.67 mm ( ±0.6 D and 0 D). CODE-V simulations with an eye relief of 30 mm validated the depth distribution. The switchable focal conditions corresponding to each of the nine depth planes, enabled by the bi-stacked QWP GPL module, are summarized in Table 1. These result from the combinatorial wavefront modulation achieved by the bi-stack configuration, which can be understood based on the polarization evolution through each optical layer within a single module unit, as detailed in Fig. 4a, and the subsequent polarization selection that yields triple-state wavefront modulation in each module. Figure 6 plots the mismatch between real and virtual depths in diopters and meters, showing that all depths fall within the 0.3 D comfort zone, spanning from 24.27 cm to infinity. Additional analysis of depth mismatches and their conversion into diopters is provided in Supplementary Section 3. The transition points between adjacent planes, represented as intersections in Fig. 6, confirm that the minimum depth mismatch remains below 0.3 D^9,11,12^: 0.3, 0.17, 0.19, 0.18, 0.21, 0.22, 0.26, 0.28, and 0.3 D from depth 1 to 9, respectively. These values ensure depth continuity within the accommodation range. In the proposed bi-stacked QWP GPL module, maintaining alignment tolerances between optical elements is essential to ensuring that the system exhibits the intended focal-switching characteristics. Supplementary Section 4 presents a detailed analysis of how variations in the parameter *d*_1_ affect the locations of the virtual image depth planes. This analysis confirms that VAC-free viewing across the entire perceptible depth range (from 25 cm to optical infinity) is achievable when *d*_1_ maintained within the range of 76.59 mm (the design condition) to 76.76 mm.

**Table 1:** Operated lens mode of two QWP GPLs and virtual image plane depth positions for optimized nine-depth virtual image formation

	Lens mode QWP GPL_1_	Lens mode QWP GPL_2_	Depth position [D]	Depth position [mm]
Depth 1	−${f}_{{{QWP\; GPL}}_{1}}$	$-{f}_{{{QWP\; GPL}}_{2}}$	3.82	261.80
Depth 2	−${f}_{{{QWP\; GPL}}_{1}}$	∞	3.46	288.61
Depth 3	−${f}_{{{QWP\; GPL}}_{1}}$	+${f}_{{{QWP\; GPL}}_{2}}$	3.09	323.78
Depth 4	∞	$-{f}_{{{QWP\; GPL}}_{2}}$	2.72	367.87
Depth 5	∞	∞	2.29	435.91
Depth 6	∞	+${f}_{{{QWP\; GPL}}_{2}}$	1.84	543.13
Depth 7	+${f}_{{{QWP\; GPL}}_{1}}$	−${f}_{{{QWP\; GPL}}_{2}}$	1.37	727.41
Depth 8	+${f}_{{{QWP\; GPL}}_{1}}$	∞	0.86	1167.28
Depth 9	+${f}_{{{QWP\; GPL}}_{1}}$	+${f}_{{{QWP\; GPL}}_{2}}$	0.30	3334.25

This design demonstrates the ability of the QWP GPL module to create nine finely spaced, switchable focal planes across the full perceivable depth range—effectively resolving VAC. Supplementary Section 5 further explains that at least seven varifocal steps are required to meet comfort zone criteria across a 25 cm–infinity range. While HWP GPL-based architectures would require three layers (2³ = 8) to approach this resolution, only two QWP GPL modules (3² = 9) are needed, offering a thinner, more efficient solution for depth-resolved XR imaging.

### Nine-depth switchable XR imaging system

**Fig. 7:**
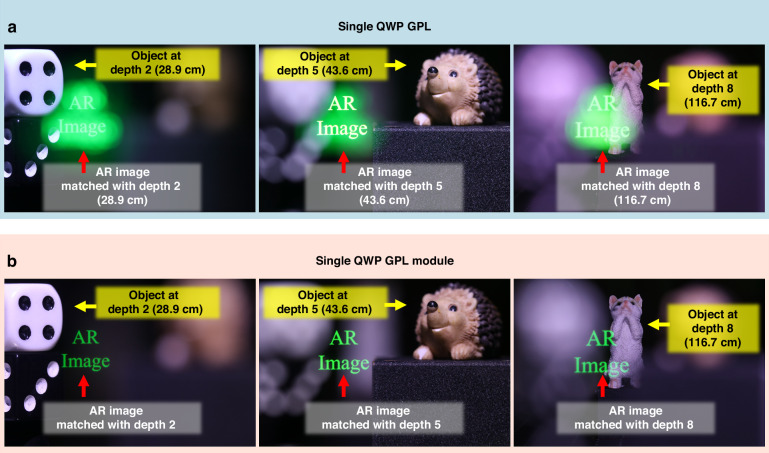
Experimental results of the XR imaging system using a single QWP GPL module. **a** Overlaid virtual images formed simultaneously at three different depth planes (28.9, 43.6, and 116.7 cm) without polarization control and selection layers. **b** A single, depth-selective virtual image clearly rendered at each depth plane using polarization control and selection layers

Prior to implementing the full nine-depth configuration, we first validated the performance of a single QWP GPL in an XR imaging system. Leveraging its intrinsic triple-wavefront modulation, this setup generated virtual images at three distinct focal depths without stacking. Using previously defined parameters and excluding QWP GPL_2_, we compared two cases: (i) a single QWP GPL_1_ without any polarization control and (ii) the same lens with integrated polarization control and selection units. The resulting AR images are shown in Fig. 7a, b. In the absence of polarization filtering (Fig. 7a), all three depth planes were simultaneously displayed, resulting in blurred virtual images due to image overlap. This confirmed the co-generation of +1^st^, −1^st^, and 0^th^ order wavefronts by the single QWP GPL. In contrast, when polarization control and selection units were added (Fig. 7b), each wavefront was selectively chosen, producing a single sharp virtual image at a desired depth plane. These results verified the selective modulation capability of the QWP GPL module. The detailed experimental conditions, including the layer composition and corresponding beam propagation and imaging results for the single QWP GPL module operation, are provided in Supplementary Section 6 along with Fig. S6.

**Fig. 8:**
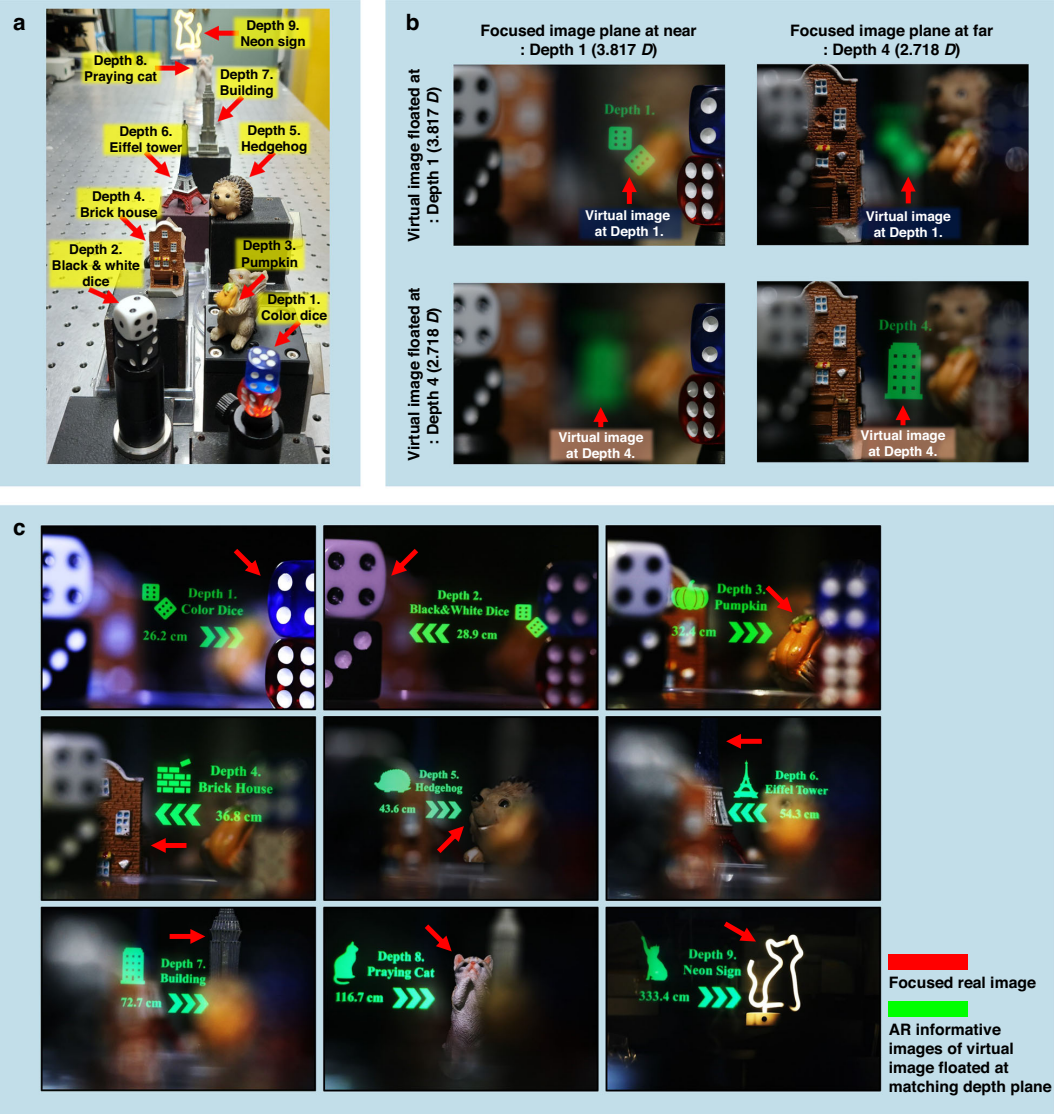
Experimental demonstration of nine-depth varifocal XR imaging with the bi-stacked QWP GPL module. **a** Arrangement of real objects used to characterize the switchable focusing function across nine virtual depth planes in the XR display optics operated by the bi-stacked QWP GPL module. **b** Captured virtual images with and without depth-plane matching, observed at real object locations corresponding to depth planes 1 (26.2 cm) and 4 (36.8 cm). **c** Image sets showing real objects alongside their corresponding XR-rendered virtual images, demonstrating accurate focus for each of the nine depth planes

Based on this confirmation, we implemented a nine-depth varifocal XR imaging system using a bi-stacked QWP GPL module (see optimized design in Fig. 5 and Table 1). Nine real objects were placed at the corresponding virtual image depths—ranging from 26.2 to 333.4 cm (depths 1 to 9)—and a Canon EOS 80D DSLR camera captured images with settings of f/4.5, ISO 3200, and 1/30 s exposure. The experimental setup is illustrated in Fig. 8a. The measured field of view (FoV) gradually increased from 10.03° to 12.91° across depths 1 to 9, as detailed in Supplementary Section 7. Under the experimentally fixed eye-relief condition of 30 mm, the effective eyebox size showed a modest decrease from 1.808 cm at depth 1 to 1.206 cm at depth 9, supporting a minimum eyebox size of 1.206 cm across all focal states. This depth-consistent eyebox tolerance ensures robust visual performance during moderate eye movements, as further supported by simulation analysis provided in Supplementary Section 8.

To evaluate the impact of VAC, we conducted a depth mismatch test between depth 1 (3.817 D) and depth 4 (2.718 D), as illustrated in Fig. 8b. At depth 1, both the green-colored virtual dice image and the colored dice object appeared in focus, while the building real object located at depth 4 was blurred. When the focal plane was adjusted to depth 4, the virtual image and real object at that depth position became sharp, whereas the dice at depth 1 appeared out of focus. The dioptric separation between these two planes was 1.1 D—well beyond the 0.3 D comfort zone threshold—demonstrating a depth mismatch condition that induces VAC.

Figure 8c presents the complete nine-depth XR imaging result, in which the virtual image planes accurately aligned with their corresponding real object positions, while maintaining inter-plane diopter differences below 0.3 D. The polarization switching configurations of the four S-HWPs, the resulting focal power states of QWP GPL_1_ and QWP GPL_2_, and both simulated and experimentally verified depth planes for all nine varifocal configurations are detailed in Supplementary Section 9 and Table S1. These results validate the varifocal accuracy of the bi-stacked QWP GPL module and its effectiveness in mitigating VAC across the full perceivable depth range—from 24.27 cm to optical infinity. Notably, this was achieved using only two stacked QWP GPL layers (3² = 9 states), whereas conventional HWP-based architectures would require at least three layers to provide a similar number of focal steps. These findings demonstrate the feasibility and scalability of QWP GPL–based modules for realizing polarization-controlled, electronically switchable, multi-depth varifocal XR displays. Furthermore, while demonstrated here using a beam-combiner architecture, the approach is compatible with waveguide- or free-form–based XR platforms, offering promising potential for reducing the volume and weight of future wearable optical systems^50^. To characterize the operational behavior of the QWP GPL module, experiments were performed using a single-wavelength light source (λ = 532 nm) to eliminate chromatic mismatch and isolate the system’s depth-switching performance. Given that the proposed GPL structure is based on a Pancharatnam-Berry phase mechanism using uniaxial, non-chiral RM layers, it exhibits inherent chromatic aberration. A detailed evaluation of this wavelength-dependent focusing property is presented in Supplementary Section 10. For extending the QWP GPL concept to full-color imaging operation, recent advances in GP optics, such as multilayer stacking of chirality-engineered RM structures tailored for R/G/B channels^20,33^, suggest promising routes.

## Discussion

This study presents a scalable varifocal lens architecture using quarter-waveplate (QWP) geometric phase (GP) optics, offering a distinctive alternative to conventional half-waveplate (HWP)–based systems. By exploiting polarization-dependent phase modulation behavior under QWP conditions, the system enables three discrete wavefront states—focusing, defocusing, and non-modulated transmission—from a single optical layer. This principle was analytically supported using the Poincaré sphere framework, offering insight into the polarization-switchable wavefront modulation behavior. The ability to support triple-state wavefront control allows QWP GPLs to achieve up to 3^n^ focal states in *n*-layer stacking configurations, significantly surpassing the binary 2^n^ transitions of HWP GPLs. In this work, only two stacked QWP GPL layers were required to implement nine distinct focal states, whereas at least three layers would be necessary in conventional HWP-based designs to achieve comparable depth resolution. This improvement not only reduces optical thickness but also mitigates interlayer alignment complexity and cumulative aberrations, which are increasing with additional GPL layers. The ability to preserve high optical quality while reducing system-level complexity is especially advantageous for precision imaging systems that rely on dynamic depth scanning and tunable focusing capabilities. While this study successfully demonstrates how replacing conventional HWP-based designs with QWP-based GP lenses can resolve the VAC in XR systems while improving form factor, the utility of this QWP-based approach is by no means limited to varifocal modules. For example, this triplet wavefront modulation strategy holds significant promise for addressing the inherently limited eyebox problem in (holographic) XR displays that is a critical challenge for practical deployment. By implementing QWP-based GPHs with prism-type phase profiles, the system form factor for active-switching beam steering can be significantly reduced without sacrificing optical function. More importantly, the triple-state wavefront modulation enabled by the QWP-based GP prism design would allow dynamic eyebox switching, facilitating seamless visual tracking and comfortable viewing across expanded eyebox zones. This functionality is essential for next-generation XR displays to accommodate natural eye movements and to support adaptive rendering via eye-tracking systems.

A key benefit of the proposed architecture lies in its ability to deliver vergence–accommodation conflict (VAC)-free imaging over the full perceivable depth range. Unlike many previous approaches that offer sparse focal planes or fail to meet perceptual comfort criteria, this system achieves dense, nine-step focal transitions under a strict 0.3 diopter spacing condition. This design threshold was successfully maintained from 24.27 cm to optical infinity. This performance is particularly relevant for near-field, interactive XR applications where frequent focus shifts occur within the user’s arm’s reach—precisely where the human visual system is most sensitive to depth cue mismatches.

The system’s fully electronic switching capability, realized via integrated switchable half-waveplates (S-HWPs), enables rapid ( <1.0 ms) and stable modulation without the need for mechanical actuation. This response time is sufficiently fast to support seamless depth transitions across all nine focal states within a single 90 Hz display frame, thereby eliminating perceptible latency or flicker. Moreover, such high-speed switching performance is advantageous regardless of the image-rendering strategy^28^ — whether employing dynamic focus-tuned image stacking synchronized with gaze tracking or depth-sliced volumetric image rendering schemes designed to mitigate the VAC in immersive XR systems. Prior studies have reported LC-based fast-switching devices with sub-millisecond response times^18,32^, and such developments ensure that the proposed QWP GPL architecture remains fully compatible with real-time, eye-tracked XR display platforms.

Beyond XR, the high focal state scalability of QWP GPLs also offers advantages for other multi-depth imaging applications, such as optical sectioning, multi-plane microscopy, or adaptive projection systems. In such use cases, the exponential growth of depth states with only modest increases in module count—scaling as 1.5^n^ relative to HWP-based approaches—offers clear benefits for systems requiring numerous focal adjustments without excessive optical path length or aberration accumulation. Furthermore, the QWP-based wavefront modulation principle introduced in this work is not limited to LC-based GPL implementations but is also inherently compatible with Pancharatnam–Berry (PB) phase-based metasurfaces, which have shown strong potential for ultrathin, multifunctional optical platforms. By applying the QWP-GP concept to PB metalenses, multi-depth switching functionality can be achieved with fewer stacked meta-layers. This is made possible by extending the conventional bi-focal switching, typically enabled by two orthogonal input polarization states, into a triple-state wavefront modulation scheme that incorporates focusing, defocusing, and non-focusing (infinity) states.

In summary, the QWP GPL module provides an electronically switchable, polarization-controlled, multi-depth varifocal platform with enhanced scalability, reduced aberration buildup, and improved compatibility with thin-form optics. These properties establish it as a promising solution for XR and other advanced optical systems requiring compact, precise, and perceptually optimized depth control. In future work, by optimizing the chirality and layer thickness conditions at each RM layer^20,33^, the uniaxial QWP GPL concept presented in this study would be expected to be extendable to achromatic, focus-tunable implementations capable of triple-state wavefront modulation for full-color VAC-free XR systems.

## Materials and Methods

The QWP GPL was fabricated using a reactive mesogen (RM) of RM257 (TCI Ltd., *n*_*e*_ = 1.678, *n*_*o*_ = 1.508, Δ*n* = 0.170 at *λ* = 550 nm) as the UV-curable retardation layer. Toluene was used as a solvent for RM dilution, and a quarter-waveplate condition (Γ = π/2) was obtained at a 10.5 wt% concentration of RM. Diluted Congo Red (azo-dye) was used as the photoalignment material with N, N-dimethylformamide (DMF) solvent. The photoalignment layer was spin-coated at 2500 rpm for 30 s in a nitrogen-filled glove box, followed by thermal baking at 120 °C for 30 min to remove solvent residues. The desired phase profile of the GPL was holographically recorded onto the photoalignment layer using polarization interference exposure setup.

**Fig. 9:**
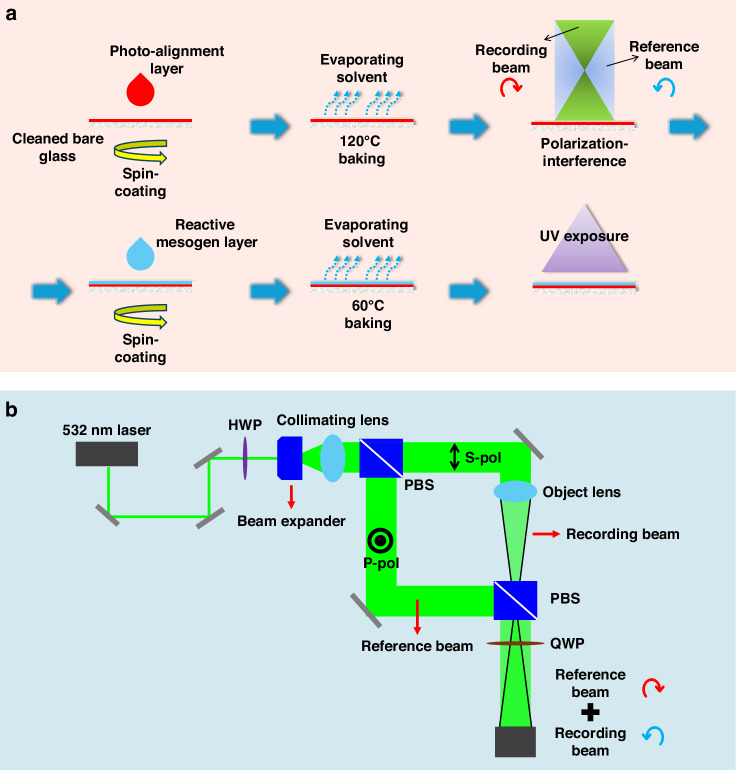
Fabrication and holographic recording of QWP GPLs. **a** Illustration of the QWP GPL fabrication process. **b** Schematic of the polarization interference–based holographic recording system used for geometric phase pattern generation

The diluted RM solution was spin-coated onto the photoaligned substrate at 2500 rpm for 30 s, followed by thermal baking at 60 °C for 1 min. The sample was then exposed to ultraviolet light (λ = 365 nm, 20 mW/cm^2^) for 2 min to polymerize and fix the RM birefringent layer through crosslinking. The resulting QWP GPL had a 1-inch aperture. The fabrication process is illustrated in Fig. 9a, and the polarization interference setup for holographic recording is shown in Fig. 9b.

**Fig. 10:**
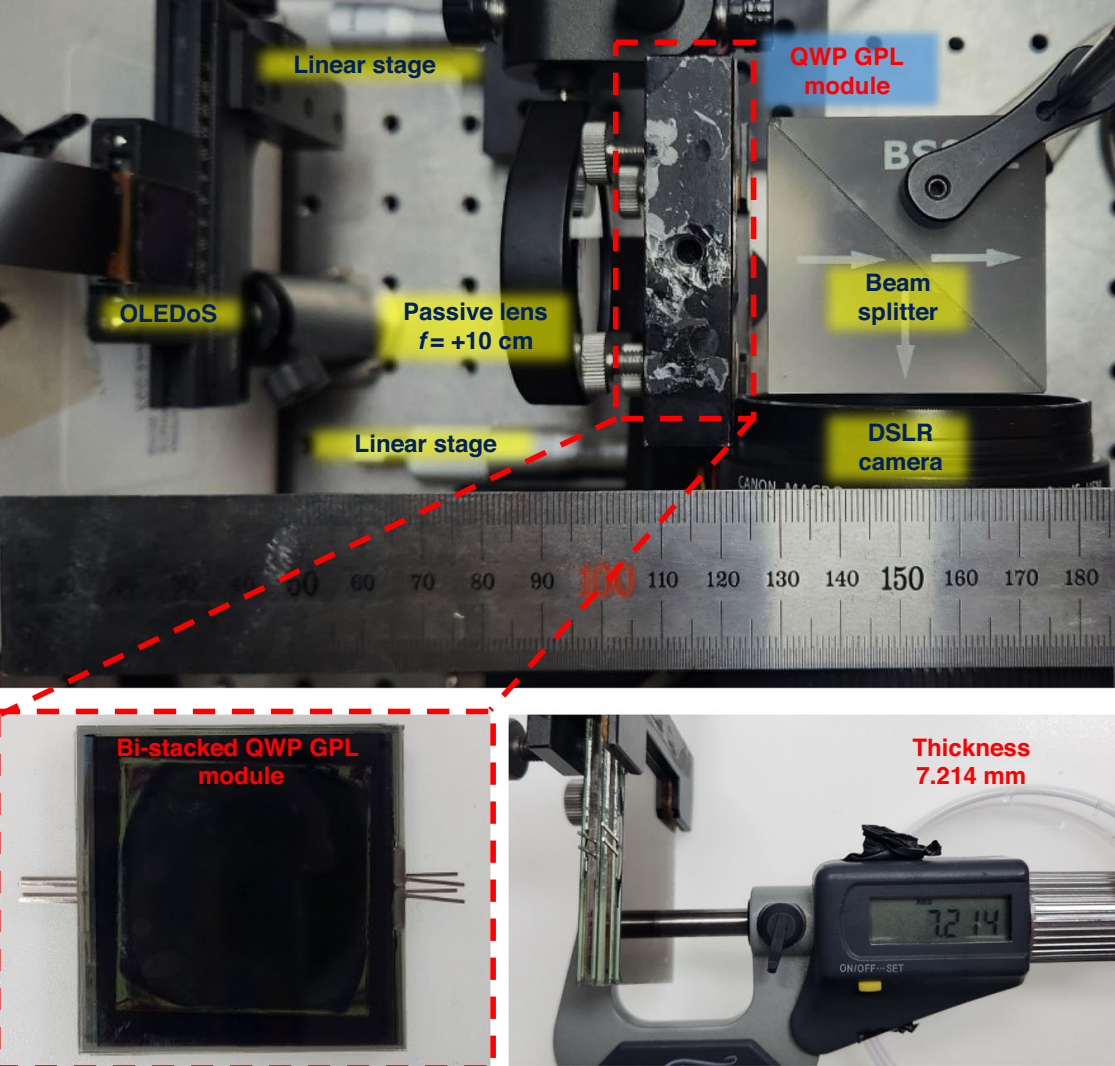
Experimental setup and QWP GPL module. Experimental setup used to evaluate the nine-depth varifocal XR imaging system, and physical thickness measurement of the fabricated bi-stacked QWP GPL module

The film-type QWP employed in the module for generating circular polarization was a commercially available QWP film (Edmund Optics, WP 140HE). For the nine-depth virtual imaging experiments, an OLED-on-silicon (OLEDoS) display panel with a 1.03-inch diagonal (SeeYA, SY103WAM01) was used. The complete AR imaging system was assembled on an optical table, as shown in Fig. 10. The total thickness of the bi-stacked QWP GPL module was measured at 7.214 mm, primarily due to the glass substrates of the S-HWPs and QWP GPLs, indicating opportunities for further volume reduction in future implementations. While the current prototype employed 0.7 mm glass substrates, replacing them with 100 μm thin-glass or film substrates is expected to reduce the overall module thickness to below 1.2 mm. Notably, the UV-cured RM layer supports a pick-up and transfer process, in which the GP layer can be detached from the fabrication substrate and laminated onto a thinner target device substrate, enabling substrate-free or ultra-thin module configurations.

The virtual image depth positions corresponding to each S-HWP switching configuration are summarized in Table S1. The four S-HWPs operate independently, yielding 16 ( = 2^4^) possible polarization configurations. Among these, nine unique varifocal modes—excluding redundant combinations yielding the same sets of the focal planes—were selected to realize the depth-switchable imaging functionality (see Supplementary Section 9).

## Supplementary information

Supplementary Information

## Data Availability

The authors declare that all data supporting the findings of this study are available within the paper and its supplementary information files.
