# Supplementary material for: Multi-depth switching by triple wavefront modulation of quarter-waveplate geometric phase lenses for vergence-accommodation-matching extended reality
Source: Light Sci Appl. 2025 Sep 19;14:333. doi: 10.1038/s41377-025-02026-2 (PMC12446484; doi:10.1038/s41377-025-02026-2)
Supplement: Supplementary file 1 — Supplementary Information [file 41377_2025_2026_MOESM1_ESM.pdf]

Supplementary Information for

Multi-Depth Switching by Triple Wavefront Modulation of Quarter-Waveplate Geometric Phase Lenses for Vergence-Accommodation-Matching Extended Reality

Jung-Yeop Shin[1,†], Jae-Won Lee[1,†], Hafiz Saad Khaliq[1], Erkhembaatar Dashdavaa[2], Munkh-Uchral Erdenebat[2], Min-Seok Kim[1], Jin-Hyeok Seo[1], Young-Min Cho[1], and Hak-Rin Kim[1,2,3*]

[1] School of Electronic and Electrical Engineering, Kyungpook National University, Daegu 41566, Republic of Korea

[2] Center for Semiconductor-Specialized University, Kyungpook National University, Daegu 41566, Republic of Korea

[3] School of Electronics Engineering, Kyungpook National University, Daegu 41566, Republic of Korea

† These authors contributed equally to this work.

*Corresponding author, E-mail: rineey@knu.ac.kr, Tel: +82539507211

1. Switching Speed of S-HWP

To characterize the dynamic switching behavior of the switchable half-waveplate (S-HWP), a single quarter-waveplate (QWP) geometric phase lens (GPL) module was constructed in the following layered sequence: S-HWP, QWP film, QWP GPL, QWP film, and a second S-HWP. The S-HWP employed an electrically controlled birefringence (ECB) liquid crystal (LC) cell (X-FPM(L)-AR, LC-Tec Displays AB), specifically configured to operate under half-waveplate conditions. Electrical control was achieved via a dedicated driving unit (model: LCC-230, LC-Tec Displays AB).

To evaluate the switching speed, the S-HWP was placed between two crossed linear polarizers (LPs), as illustrated in Fig. S1a. The optical transmittance during voltage transitions was monitored in real time using a photodetector. The switching speed was defined as the time required for the transmitted intensity to change between 10% and 90% of its steady-state value, during both the rising and falling transitions of the electric field. As shown in Fig. S1b, the field-off time was measured to be 1.04 ms, while the field-on time was 0.23 ms. These results confirm the S-HWP's suitability for fast polarization switching in varifocal optical systems.

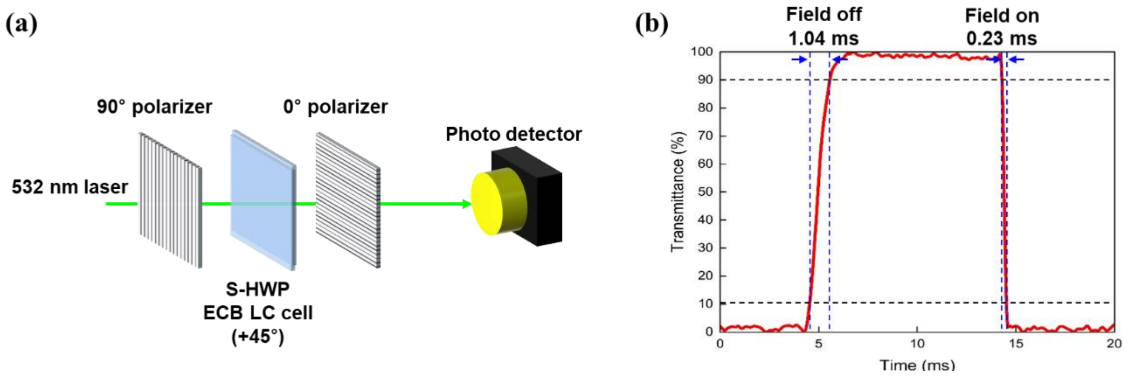

Fig. S1 (a) Schematic of the optical setup used to evaluate the switching speed of the switchable half-waveplate (S-HWP). (b) Measured transmittance curve illustrating the time-dependent response of the S-HWP during electric field switching.

2. Composition and Operation of Multi-Step Varifocal HWP GPL Module

To utilize the polarization-switching–based wavefront modulation capability of the HWP GPL, a multilayer module was constructed by stacking an electrically switchable polarization component and a fixed phase retarder, as illustrated in Fig. S2a. This single-layer module enables up to two distinct wavefront modulation states, corresponding to the +1st and -1st diffraction orders.

The HWP GPL module was assembled in the following sequence: S-HWP, film-type QWP, HWP GPL, and another film-type QWP. An LP was placed at the front of the module to convert the incident beam into a linearly polarized state. The slow axis of the QWP was oriented at 45° with respect to the linear polarization direction of the incoming beam, allowing the resulting circular polarization state incident on the HWP GPL to be dynamically switched between left- and right-handed circular polarization. This switching was controlled by the S-HWP, which incorporates an LC cell with electrically tunable birefringence.

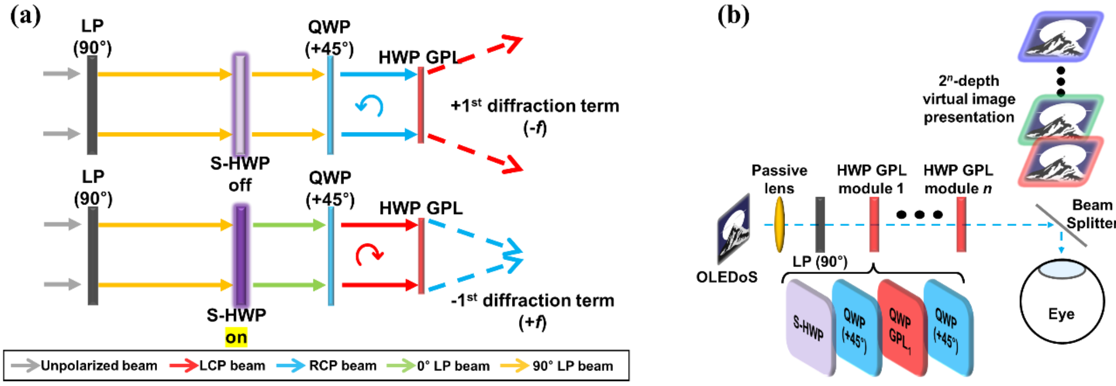

Fig. S2 (a) Schematic illustrates the composition of a single half-waveplate (HWP) geometric phase lens (GPL) element and its polarization-dependent wavefront modulation effects. (b) Schematic of a bird-bath–type extended reality (XR) imaging system implemented with an n-layer stacked HWP GPL module.

Since each HWP GPL module can selectively generate two distinct wavefronts depending on the handedness of the incident circular polarization, stacking n such modules enables up to 2^n

unique wavefront modulation states. When integrated into a bird-bath–type XR imaging system, as illustrated in Fig. S2b, this configuration facilitates the generation of virtual images at 2^n discrete depth planes under appropriately designed optical conditions. This architecture offers a compact and scalable solution for implementing multi-depth varifocal functionality using HWP-based GPL optics.

3. Depth Mismatch Verses Distance in Real-World and Comfort Zone for Varifocal XR Imaging System

This study presents a nine-depth XR imaging system capable of representing virtual image planes in both spatial distance and dioptric terms. The depth positions of each virtual image plane were validated against CODE V ray-tracing simulations to ensure consistency. Figures S3a and S3b show the spatial mismatch between the virtual image planes and the real-world scene plane, plotted in meters and diopters, respectively.

The relative positions of the virtual planes are preserved across both spatial and dioptric representations, resulting in identical dioptric differences between each virtual plane and the real object distance. As a result, the intersection points of the lines—marked as gray spots in both figures—remain fixed regardless of the axis representation. These intersection points define a set of continuous depth regions in which the accommodation demand is effectively distributed, thereby satisfying the comfort zone criterion through coverage provided by the adjacent virtual planes.

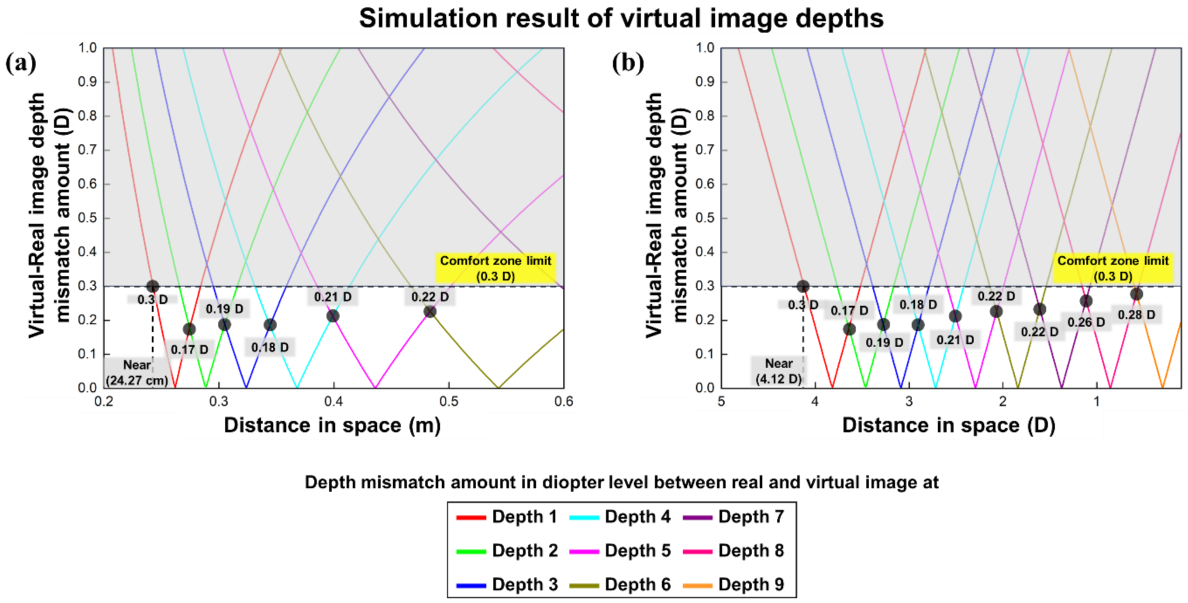

Fig. S3 (a) Depth mismatch between the virtual image plane and the real-world scene plane, expressed in meters, for distances ranging from 20 cm to 60 cm. (b) Depth mismatch expressed in diopters (5 D to 0.1 D) for a nine-depth varifocal optical system implemented with a bi-stacked QWP GPL module. Intersection points between adjacent depth mismatch curves are marked in the graph.

While Fig. S3a uses a spatial (meter-scale) representation like that in the main text, Fig. S3b provides a complementary dioptric view, where the *x*-axis is expressed in diopters. This format renders the lines visually linear, allowing for more intuitive interpretation of the accommodation profile across the depth range.

4. Mechanical Alignment Tolerance for Maintaining a VAC-Free Condition in a Nine-Step Varifocal XR Imaging System

In this supplementary section, we present the mechanical alignment tolerance required to ensure that the proposed VAC-free XR imaging system maintains vergence–accommodation consistency across its full operational depth range. The optical system is designed to limit the perceptible focal plane mismatch—interpreted through vergence and accommodation cues—to within 0.3 D over a depth range spanning from 25 cm to optical infinity.

In the proposed bi-stacked QWP GPL module, maintaining alignment tolerances between optical elements is essential to ensuring that the final system exhibits the desired focal-switching characteristics. Specifically, two types of mechanical alignment tolerances must be considered: (1) gap alignment tolerance, which governs the axial positioning of lens elements and thus directly impacts the depth accuracy and VAC performance; and (2) polarization alignment tolerance, which determines how accurately the optic axes of polarization-sensitive elements are oriented with respect to each other, affecting the intended polarization transformations and resulting wavefront modulations. Both aspects are discussed in detail in the following sections.

First, in our proposed nine-step varifocal XR imaging system, the mechanical alignment parameters that determine whether each virtual image depth plane remains within the 0.3 D comfort zone (for mitigating VAC) are the spatial gaps d_1, d_2, and d_3, as illustrated in Fig. 5b of the manuscript.

In the proposed design, the farthest virtual image depth (depth 9), positioned at 0.3 D (equivalent to 3.33 m), spans a range from 0.6 D (1.67 m) to 0 D (optical infinity). Similarly, the nearest virtual image depth (depth 1), located at 3.82 D (26.18 cm), spans a range from 4.12 D (24.27 cm) to 3.52 D (28.41 cm). Due to the overlap between these depth ranges, the system enables continuous VAC-free virtual image presentation from 24.27 cm to infinity.

Among the three spatial gap parameters d_1, d_2, and d_3, the most critical factor governing this VAC-free depth coverage is the axial distance d_l between the OLEDOS panel and the passive lens. While the nine-step varifocal depths are uniformly spaced at 0.6 D intervals—this interval remains invariant under variation in d_l. However, changes in d_l cause a global shift of all nine virtual image depths either toward the near or far field, while preserving the 0.6 D step spacing.

Fig. S4a illustrates the depth mismatch between the virtual image planes and the corresponding real-world scene planes (expressed in meters), when d_l is increased to 76.76 mm, with all other parameters fixed. In this scenario, depth 1 is located at 3.7 D, and depth 9 is at 0.18 D. Despite this shift, the system remains the 0.3 D comfort zone and therefore preserves VAC-free imaging operation across the entire depth range from 25 cm to infinity. As a result, the allowable range of d_l for VAC-free coverage is determined to be between 76.59 mm and 76.76 mm.

In contrast, Fig. S4b illustrates a case where this tolerance is exceeded. When d_l is set to 71.00 mm, the entire set of focal planes shifts toward the user, restricting the VAC-free range to approximately 20.70 cm to 1.44 m. Beyond 1.44 m, the vergence-accommodation mismatch exceeds the comfort threshold, potentially leading to VAC-induced visual discomfort.

In the bi-stacked QWP GPL module, the gap alignment tolerance issue between optical units can be summarized as follows.

- The gap parameters d_2 and d_3, which represent the distances from the passive lens to QWP GPL_1 and from QWP GPL_1 to QWP GPL_2, respectively, are designed to be significantly smaller than the focal lengths of the QWP GPLs. Under such conditions in our experiment, as described by Equations (6)–(8) in the manuscript, their influence on the final image depth positions is negligible. This aspect also means that minimizing d_2 and d_3 is desirable for reducing the sensitivity of the switchable depth formation by

the bi-stacked module to inter-element gap variations, which is also suitable for the slim form-factor of the total module.

- In contrast, d_1, the distance between the display panel and the passive lens, directly determines the absolute locations of all sets of virtual image depth planes. Any variation in d_1 induces a uniform dioptric shift across the nine focal states. Therefore, precise mechanical control of d_1 is essential to maintain the intended depth distribution. In our design, the VAC-free viewing condition, within the 0.3 D comfort zone across the perceptible range (25 cm to optical infinity), is preserved when d_1 falls within the range of 76.59 mm (the design d_1 condition) to 76.76 mm. As shown in Fig. 6, the near-depth focal planes are more closely spaced than those in the far-depth region. Consequently, the acceptable tolerance window is slightly shifted in the positive direction from the nominal design.

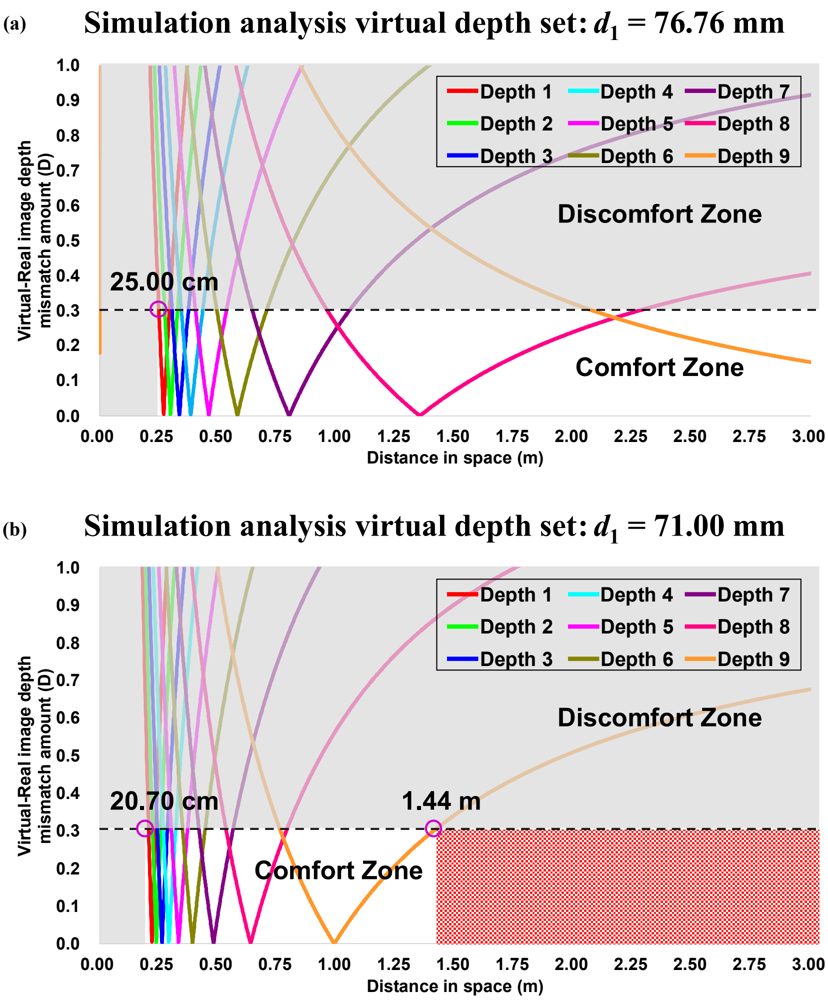

Fig. S4 Depth mismatch between the virtual image plane and the real-world scene plane, expressed in meters, for (a) $d_1 = 76.76$ mm and (b) $d_1 = 71.00$ mm.

Second, polarization alignment depends on the relative rotation angles among polarization-sensitive components such as the LP, S-HWPs, and QWP films. In our design:

- The linear polarizer (LP) is fixed at a 90°.
- Each optic axis of the S-HWP (realized using ECB-mode LC) is oriented at +45° relative to the LP, enabling polarization switching between 90° and 0° linear polarizations.
- Each optic axis of the QWP film is aligned at +45°, converting orthogonal linearly polarized beams into corresponding circularly polarized beams.

Any optic axis misalignment in these elements (e.g., due to fabrication tolerances) may prevent the incident polarization from being properly transformed into the desired circular polarization before entering the QWP GPL. This misalignment could compromise the dual or triple virtual images on each different depth positions, potentially resulting in ghost images at unintended depth planes.

Lastly, while the optical axis alignment between the two QWP GPL elements does not directly affect the 0.3 D comfort zone criterion, it remains a critical factor influencing both image quality and eyebox stability. As in any imaging system, axial misalignment between lens elements can lead to image degradation or shifts in the eyebox position. In this respect, it is worth emphasizing that our proposed system offers a distinct advantage over conventional HWP GPL-based varifocal modules: by enabling a greater number of focal states with fewer physical optical layers, the QWP GPL architecture reduces the number of alignment-critical interfaces, thereby mitigating cumulative alignment errors during assembly.

Despite the clear advantages of the QWP GPL stack module over the HWP GPL stack module in terms of reducing optical alignment issues, it is nonetheless advisable to employ active alignment techniques during the assembly process. This is essential to minimize deviations from the design specifications, particularly with respect to unit-to-unit gap tolerances, optical axis misalignment, and axial centering tolerances between lens elements.

5. Ideal Depth Plane Design Under Comfort Zone Criteria

The comfort zone is defined as the depth range within the human eye's depth of field (DoF) surrounding a virtual image plane. It is quantitatively expressed as the virtual image depth ± the eye's DoF, measured in diopters. For example, a virtual image depth positioned at 0.3 D (3.33 m) yields a comfort zone spanning from 0.6 D (1.67 m) to 0 D (infinity).

To achieve a continuous and visually comfortable depth coverage, the comfort zones of adjacent virtual image planes must overlap. Accordingly, the optical design requires at least seven depth-tunable virtual planes to fully span the perceivable range of a typical adult viewer, from 25 cm (4.0 D) to infinity (0 D). The selected dioptric positions for the virtual planes are: 0.3 D (3.33 m), 0.9 D (1.11 m), 1.5 D (0.67 m), 2.1 D (0.48 m), 2.7 D (0.37 m), 3.3 D (0.30 m), and 3.9 D (0.26 m), as illustrated in Fig. S5.

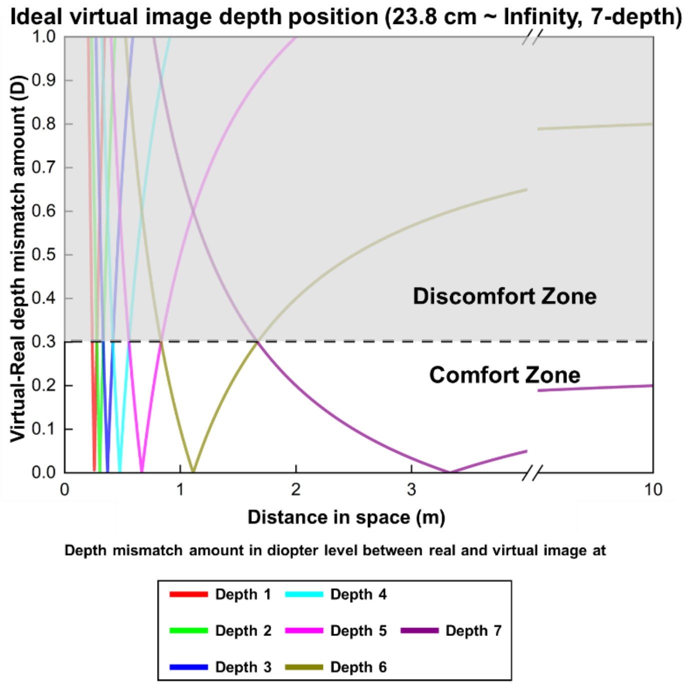

Fig. S5 Ideal depth plane design satisfying the accommodation comfort zone criterion (< 0.3 D). The comfort zone is extended from 23.8 cm to infinity by incrementally designing adjacent virtual depth planes, ensuring that the entire perceivable depth range remains within the depth of field (DoF) of the human visual system.

Each line in Fig. S5 represents the dioptric depth mismatch between the virtual image plane and real-world scene distances. All lines intersect at the 0.3 D level, illustrating that the entire perceivable depth range—from infinity (0 D) to 4.2 D (0.24 m)—can be fully encompassed within the human eye's DoF. This design ensures that the vergence–accommodation mismatch remains confined within the comfort zone across all target depths, enabling visually comfortable XR experiences over the full near-to-far range.

6. Experimental Setup and AR Imaging Results Using a Single QWP GPL and a QWP GPL Module

Figure 7 in the main text presents the experimental demonstration of virtual image formation in an XR imaging system using either a single QWP GPL or a QWP GPL module composed of multiple polarization-selective elements. The display device was an OLEDoS panel with a 1.03-inch diagonal (SY103WAM01, SeeYA), and a plano-convex lens with a focal length of +10 cm (LA1050, Thorlabs) served as the passive focusing optic. A linear polarizer (LP) was placed in the optical path to transmit only light polarized at 90°.

As shown in Fig. S6a, when a single QWP GPL is integrated into the XR system, the incident linearly polarized light produces a triple-wavefront effect. This leads to the simultaneous formation of three virtual images at different focal depths, corresponding to depth planes 2, 5, and 8, as defined in the main text. However, since all three wavefronts are generated concurrently, only the image at the observer's accommodation depth appears sharp, while the others exhibit significant defocus blur. This overlap limits the system’s ability to render clear, depth-resolved information.

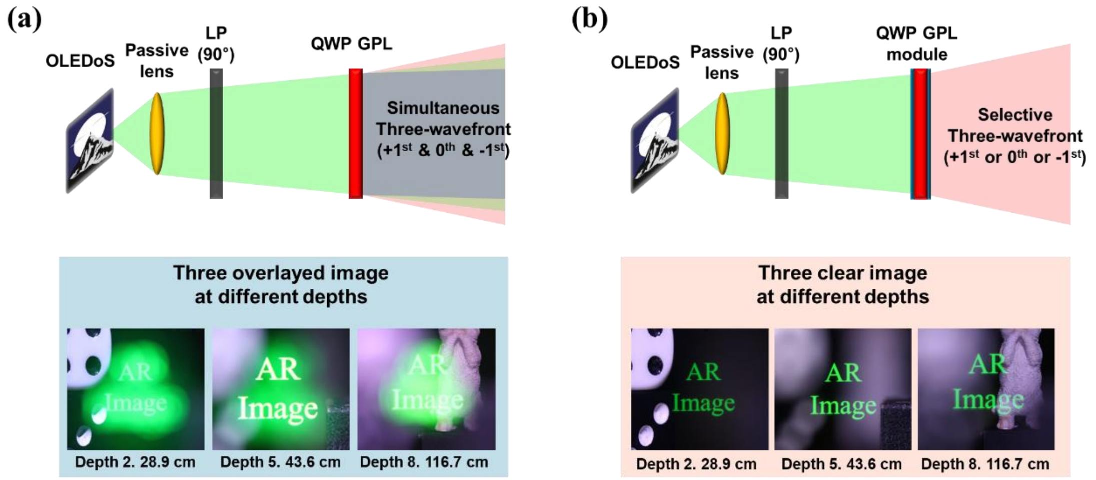

Fig. S6 Experimental setup and magnified augmented reality (AR) images obtained using (a) a single quarter-waveplate geometric phase lens (QWP GPL) and (b) a QWP GPL module with polarization control and selection layers.

To overcome this limitation, a QWP GPL module was developed with a layered configuration incorporating dynamic polarization-switching components. As demonstrated in Fig. S6b, this modular structure allows for independent selection of one of the three wavefronts, enabling virtual image formation at a specific target depth without overlapping. This selective addressing mechanism improves visual clarity and provides a practical solution for rendering focal depth in XR systems with minimal optical complexity.

7. Field of View Evaluation for Nine-Depth Virtual Image Planes

The field of view (FoV) of virtual images generated by the nine-depth XR imaging system was evaluated as shown in Fig. S7. An OLEDoS panel (SY103WAM01, SeeYA) served as the display, where an arrow-shaped image labeled "FoV" was projected diagonally across the full panel area. The QWP GPL module was used to form virtual images at nine discrete depth planes, and the maximum diagonal length of each virtual image was measured at the corresponding focal depth. The measured diagonal sizes for depth levels 1 through 9 were 4.59, 5.53, 6.39, 7.35, 9.11, 11.46, 15.69, 25.93, and 75.41 cm, respectively. A scalable ruler was placed at the focused image depth during image capture to ensure accurate measurement. The FoV at each virtual depth plane (indexed by k, where k = 1 to 9) was calculated using the following geometric relation:

$$\mathrm{FoV}\ (k) = 2 \cdot \tan^{-1}\left(\frac{L_k}{2v_k}\right) \tag{1}$$

where L_k denotes the measured diagonal length of the virtual image at depth k, and v_k represents the corresponding virtual image distance. The resulting FoV values for depth planes 1 to 9 were calculated as 10.03°, 10.93°, 11.26°, 11.41°, 11.93°, 12.04°, 12.32°, 12.68°, and 12.91°, respectively.

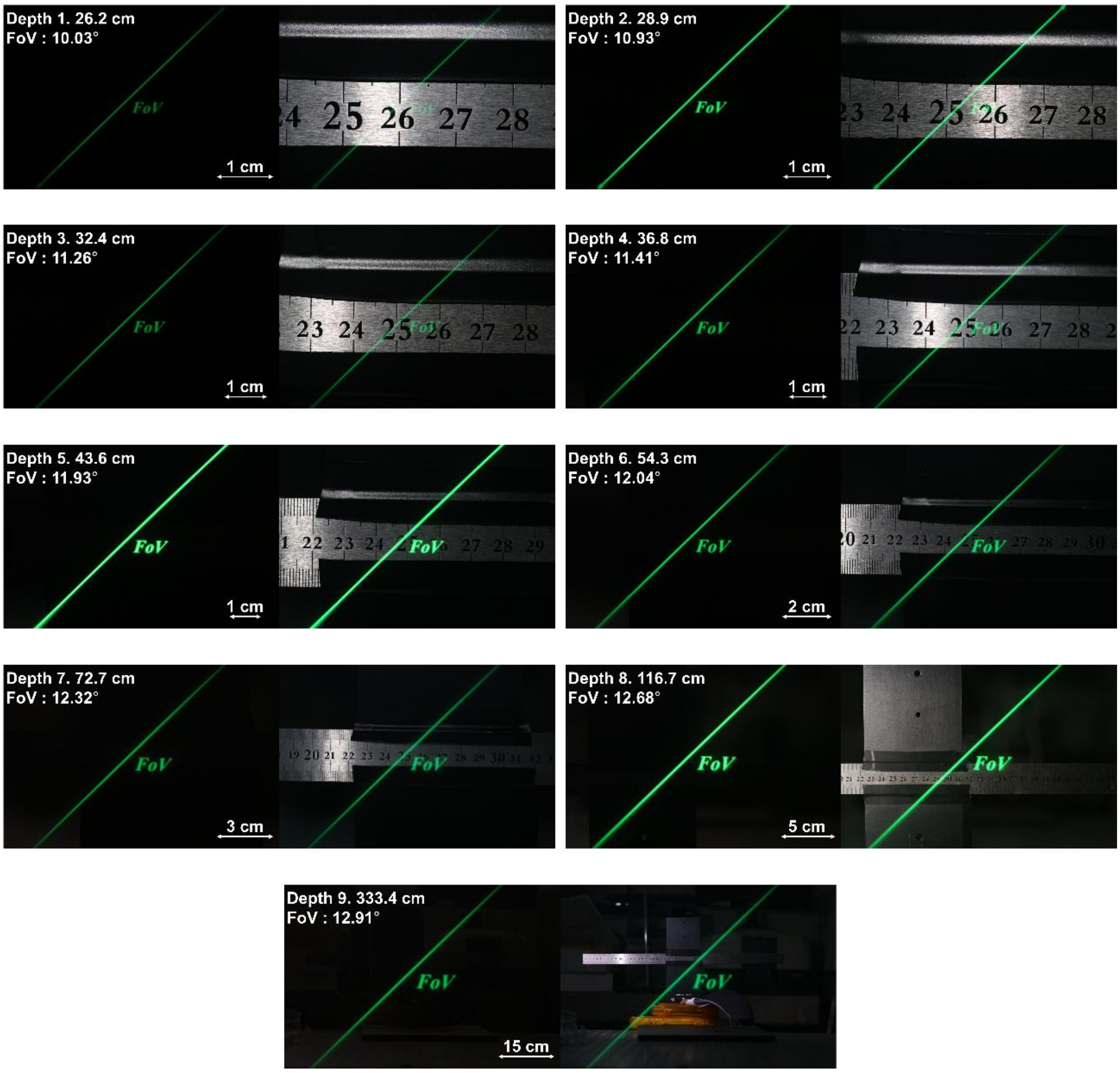

Fig. S7 Captured arrow-shaped virtual images spanning the maximum diagonal extent at each of the nine optimized virtual depth planes, used for evaluating the field of view (FoV).

8. Analysis of Eyebox Size in a Nine-Step Varifocal XR optical system

Based on the configuration illustrated in Fig. 5b, we implemented a nine-step varifocal optical system incorporating a bi-stacked QWP GPL module. The system was designed with a fixed eye relief of 30 mm. The corresponding virtual image planes for depth levels 1 through 9 were located at 3.82 D, 3.46 D, 3.09 D, 2.72 D, 2.29 D, 1.84 D, 1.37 D, 0.86 D, and 0.30 D, respectively. Although the optimal eye relief for maximizing the eyebox may vary depending on the virtual image depth, practical usage conditions in XR optical systems typically require a fixed eye relief—defined as the distance from the user's eye to the final optical surface of the headset. Consequently, the eyebox size inherently varies with focal depth due to varifocal modulation.

To evaluate this depth-dependent variation in eyebox size, we conducted simulations for each of the nine depth-switching states using the designed varifocal optical system, as shown in Fig. S8a. The resulting eyebox sizes for depth levels 1 through 9 were 1.808 cm, 1.790 cm, 1.770 cm, 1.691 cm, 1.604 cm, 1.505 cm, 1.396 cm, 1.294 cm, and 1.206 cm, respectively. These values are plotted as a function of virtual image depth (in diopters) in Fig. S8b. The plot reveals a monotonic decrease in eyebox size as the virtual image shifts from near to far depth, indicating that the largest eyebox is achieved when the focal plane is nearest to the user (depth 1), and the smallest when projected to the farthest distance (depth 9). Nevertheless, this consistent depth-dependent eyebox tolerance ensures robust visual performance during moderate eye movements, with a minimum maintained eyebox size of 1.206 cm across all focal states.

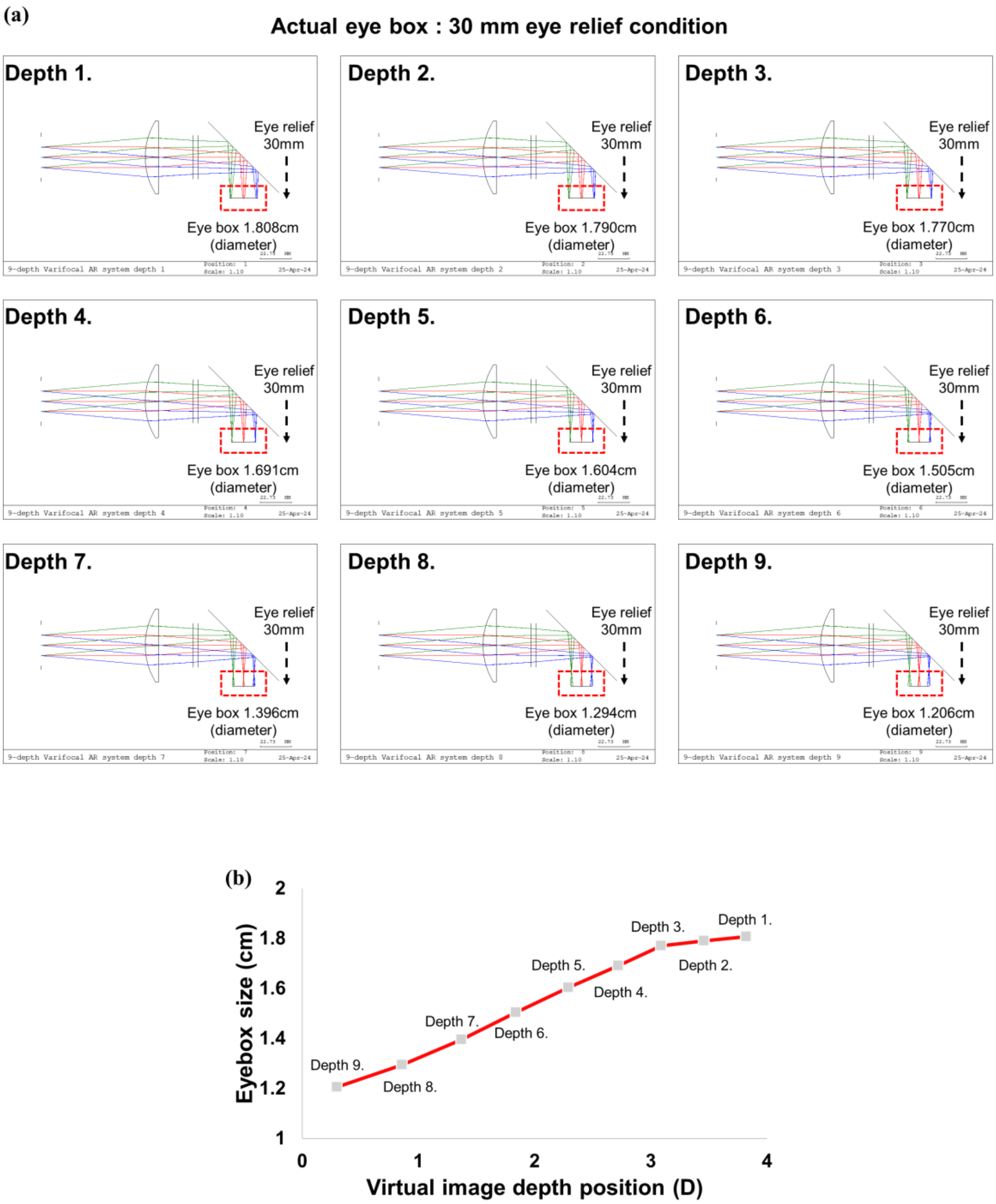

Fig. S8 (a) Simulation analysis of eyebox changes for each of the nine depth-switching states under the fixed eye relief of 30 mm in the XR optical system employing a bi-stacked QWP GPL configuration. (b) Graph of eyebox size according to the virtual image depth position in diopter level.

9. Varifocal Operation of the QWP GPL Module Based on the S-HWP Operation

The proposed QWP GPL module enables selective generation of wavefronts by controlling the handedness of circularly polarized light incident on each QWP GPL element. This polarization switching is realized through the field-on and field-off states of the integrated S-HWPs. The bi-stacked module incorporates four S-HWP layers, which govern the varifocal functionality by modulating the polarization states entering and exiting the two QWP GPLs. Each QWP GPL in the module can simulate lensing behavior with positive, negative, or effectively infinite focal lengths, depending on the operational configuration of the associated S-HWPs. By combining the three wavefront modulation states of each QWP GPL, the module can theoretically produce up to $3^2 = 9$ distinct virtual focal depths. Table S1 summarizes the simulated and experimentally verified positions of the nine virtual image planes generated by the XR imaging system using this configuration.

Table S1. Virtual image plane depth positions corresponding to the nine optimized focal states, determined by S-HWP operations and two QWP GPL configurations.

Depth	Switchable HWP				Operated lens mode		Virtual image depth position [D]	
	$S\text{-}HWP_1$ [V_{pp}]	$S\text{-}HWP_2$ [V_{pp}]	$S\text{-}HWP_3$ [V_{pp}]	$S\text{-}HWP_4$ [V_{pp}]	$QWP\ GPL_1$	$QWP\ GPL_2$	Simulated	Experimental
1	12	0	0	0	$-f_{QWP\ GPL_1}$	$-f_{QWP\ GPL_2}$	3.82	3.83
2	12	0	0	12	$-f_{QWP\ GPL_1}$	∞	3.46	3.47
3	12	0	12	12	$-f_{QWP\ GPL_1}$	$+f_{QWP\ GPL_2}$	3.09	3.10
4	0	0	0	0	∞	$-f_{QWP\ GPL_2}$	2.72	2.72
5	0	0	0	12	∞	∞	2.29	2.30
6	0	0	12	12	∞	$+f_{QWP\ GPL_2}$	1.84	1.84
7	0	12	0	0	$+f_{QWP\ GPL_1}$	$-f_{QWP\ GPL_2}$	1.37	1.38
8	0	12	0	12	$+f_{QWP\ GPL_1}$	∞	0.86	0.86
9	0	12	12	12	$+f_{QWP\ GPL_1}$	$+f_{QWP\ GPL_2}$	0.30	0.30

10. Chromatic Aberration in Nine-Step Varifocal AR Optics with Bi-Stacked QWP GPL Module

The principle of wavefront modulation based on the Pancharatnam–Berry phase relies on the spatially varying optical axis orientation in anisotropic retardation media, which induces a geometric phase through relative phase delay. When implemented using uniaxial birefringent materials without chirality, such as in our non-chiral RM alignment, this modulation becomes intrinsically wavelength-dependent, resulting in chromatic aberration in geometric phase lenses (GPLs). In our fabricated GPLs, all designed focal lengths were calibrated at the design wavelength, λ_d = 532 nm. Because the proposed GPL structure is based on the Pancharatnam–Berry phase using non-chiral RM alignment, it inherently exhibits chromatic focal dispersion. Although the present study primarily aims to demonstrate the functional and structural advantages of a QWP-based GPL configuration—particularly its capability for triple-state wavefront modulation and its potential to reduce the overall optical module form factor compared to conventional HWP-based designs, for the benefit of readers, we provide here a detailed characterization of the chromatic aberration behavior observed in the fabricated QWP-GPL module.

When light of a different wavelength λ_i propagates through the GPL, the effective focal length f_i is given by the following relation:

$$f_i = (\lambda_d/\lambda_i) f_d$$

This chromatic dispersion results in wavelength-dependent focal shifts, where the red (R), green (G), and blue (B) components form focal planes at different depths during full-color imaging. Specifically, compared to the designed focal length at the green wavelength, the focal length becomes relatively shorter for red light and longer for blue light. Consequently, when the system is focused to a single depth, the images from the other color channels become defocused, manifesting as chromatic blur. This phenomenon is inherent not only in

conventional HWP-based GPLs but also in the QWP-based GPLs presented here, as both rely on uniaxial structures with spatially modulated optic axes in the absence of chirality.

We experimentally captured the chromatic focal shift using the nine-step varifocal optical system incorporating a bi-stacked QWP GPL module, as shown in Fig. S9. RGB focal planes were recorded at three representative depths: Depth 1, Depth 5, and Depth 9. At Depth 5 configured such that the two QWP GPL modules together produce an effective focal length approaching infinity as shown in Table 1, the R, G, and B channels were focused at nearly identical depths of 2.32 D, 2.30 D, and 2.28 D, respectively. This result indicates that the chromatic focal shift at this depth arises solely from the dispersion of the passive lens and remains minimal.

However, we also analyzed the Depth 1 and Depth 9 conditions, in which the focal powers of the QWP GPL modules were set to their maximum positive and negative values, respectively—representing the configurations with the most pronounced chromatic aberration due to the strongest wavefront modulation in the GPLs. At Depth 1, QWP GPL_1 and QWP GPL_2 provided focal powers of –1.8 D and –0.6 D at 532 nm, resulting in a sharp green focus at 3.83 D. For other wavelengths, the focal powers deviated from the designed condition: at 660 nm (red), the corresponding powers were –2.23 D and –0.74 D, while at 457 nm (blue), they were –1.55 D and –0.51 D. In the case of Depth 9, the signs of these focal powers were simply reversed. This wavelength-dependent variation in focal power results in color-dependent focal plane shifts across the RGB channels, as visually characterized in Fig. S9.

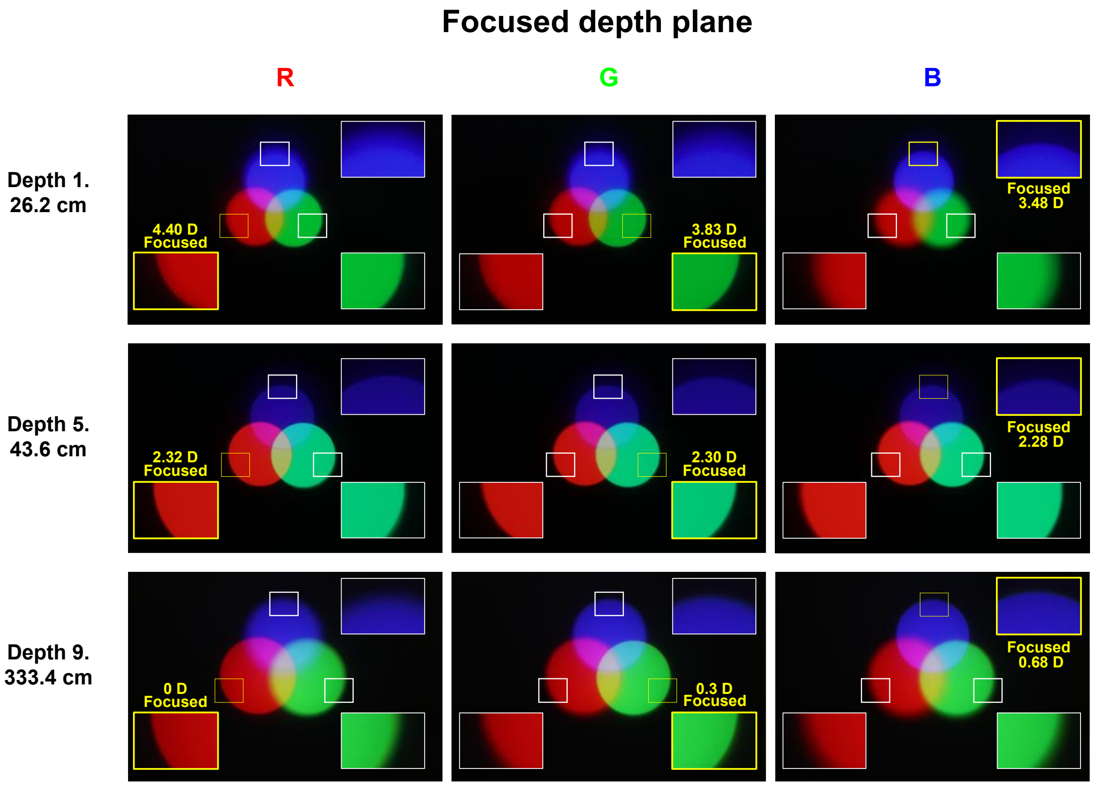

Fig. S9 Wavelength-dependent focal plane positions (R, G, B) at depth position 1, 5, and 9 in the XR optical system employing a bi-stacked QWP GPL configuration.

In GPLs based on the Pancharatnam–Berry phase using uniaxial structures without chirality, the focusing behavior inherently exhibits wavelength-dependent focusing efficiency variation as well as wavelength-dependent focal shift. Specifically, the geometric phase modulation efficiency varies with the incident wavelength due to chromatic retardation deviations from the designed retardation condition ($\Gamma=2\pi\Delta nd/\lambda$). For RM-based GPLs, the theoretical maximum diffraction efficiency reaches 100% when the phase retardation $\Gamma = \pi$ for the HWP condition. However, at wavelengths deviating from the design wavelength, residual 0th-order non-diffracted wavefronts are generated, which can lead to depth cross-talk in the HWP-GPL module. In contrast, the proposed QWP-GPL module is designed to achieve a phase retardation of $\Gamma = \pi/2$, wherein the focusing and non-focusing modes ideally form with a 1:1 intensity ratio. Although deviations from the target wavelength band cause intensity ratio imbalance between the 1st order and 0th order diffraction terms, the QWP-based module scheme still enables the

respective wavefronts to function as either focusing or non-focusing modes, thereby facilitating triple-wavefront modulation. Therefore, from another perspective, the QWP-GPL stacked module may offer inherently better depth-selective imaging performance in our demonstration of single-color depth switching even under the practical limitations in precisely achieving the ideal half- and quarter-wave retardation conditions in the fabricated HWP and QWP GPLs, respectively.

Table S2 summarizes the measured diffraction efficiencies of both HWP and QWP GPLs at RGB wavelengths under convex-lens mode operation, achieved through incident circular polarization control. Both devices were fabricated under identical processing conditions using the same RM material with identical birefringence (Δn), but with different layer thicknesses optimized to satisfy their respective retardation conditions. According to the retardation relation ($\Gamma = 2\pi\Delta nd/\lambda$), the RM layer in the QWP GPL is designed to be half as thick as that in the HWP GPL. This reduced thickness increases the wavelength sensitivity of the retardation deviating from the designed retardation, resulting in higher variation in focusing efficiency across the visible spectrum. Consequently, from the perspective of broadband imaging efficiency, the development of achromatic GPL, through strategies such as chirality-engineered and/or multi-stacked structures, is more critically required for QWP GPLs than for their HWP GPLs.

Table S2. Comparison of wavelength-dependent focusing efficiencies between HWP and QWP GPLs, each designed with optimal retardation conditions at the green wavelength.

		Modulation Efficiency (%)		
Wavelength		R (660nm)	G (532nm)	B (457nm)
HWP GPL	1st (focusing mode)	63.57	94.19	93.25
	0th (depth crosstalk noise)	36.43	5.81	6.75
QWP GPL	1st (focusing mode)	33.75	45.24	67.40
	0th (Non-focusing mode)	66.25	54.76	32.60

Nevertheless, a comparative MTF analysis using captured USAF resolution target images (Fig. S10) confirms that the QWP GPL module maintains imaging quality comparable to the HWP-based counterpart. At spatial frequencies of 1, 2, and 4 lp mm-1, the measured MTF values were 0.396, 0.243, and 0.101 for the HWP GPL module, and 0.413, 0.257, and 0.101 for the QWP GPL module, respectively. These results demonstrate that the QWP GPL module does not exhibit any degradation in imaging performance, despite its inherently lower theoretical diffraction efficiency.

This robustness is attributed to the incorporation of a polarization-selection layer in the QWP GPL module, which actively filters either the diffracted or non-diffracted wavefront components to realize triple-state modulation. While the amplitude of the modulated wavefront may vary with wavelength, this selection mechanism effectively suppresses noise components—particularly, it eliminates image-doubling artifacts caused by crosstalk between focal states. As a result, the overall imaging fidelity remains consistent between the two GPL designs.

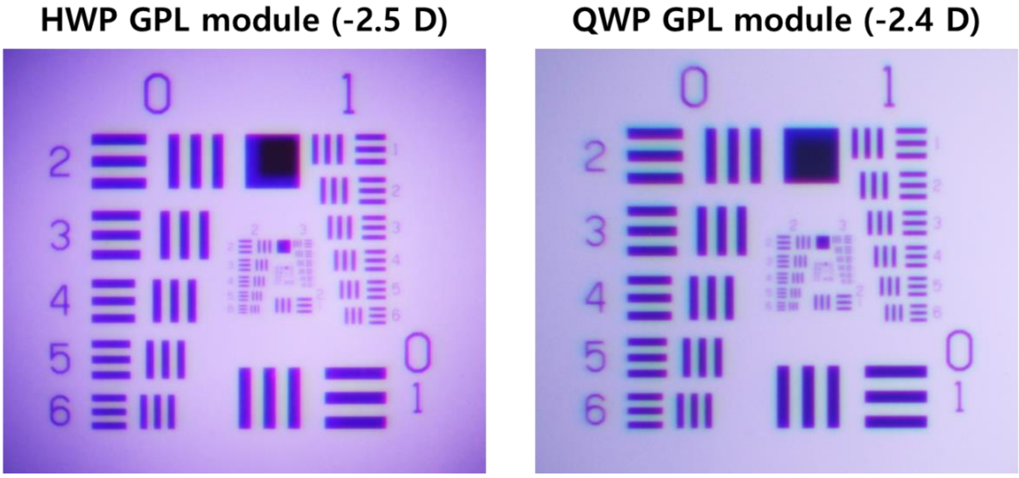

Fig. S10 USAF resolution target images, captured for image resolution comparison between the HWP and QWP GPLs, where white light illumination is employed for characterizing light source.

Of course, further enhancements in imaging performance for both GPL types will benefit from improved RM alignment techniques. More fundamentally, as previously discussed, achieving

achromatic operation—while beyond the scope of the present study—is essential for the practical realization of full-color, VAC-free XR systems. Building upon previously proposed strategies for HWP-based GPLs, such as chirality-engineered RM stacks tailored for R/G/B channels, similar approaches can be adapted to QWP-based architectures. Crucially, even under such RGB-selective stacking schemes, the triple wavefront modulation strategy demonstrated in this work retains a key advantage: it enables exponential scaling of focal states (3^n) using fewer physical layers, thereby reducing overall module thickness and optical complexity. Realizing this capability under achromatic conditions will require precise optimization of both chirality and layer thickness in each RM layer—an active area of ongoing research in our group.
